# Graph Representation Forecasting of Patient's Medical Conditions: Toward a Digital Twin

**DOI:** 10.3389/fgene.2021.652907

**Published:** 2021-09-16

**Authors:** Pietro Barbiero, Ramon Viñas Torné, Pietro Lió

**Affiliations:** Department of Computer Science and Technology, University of Cambridge, Cambridge, United Kingdom

**Keywords:** digital twin, generative adversarial networks, monitoring, graph representation learning, precision medicine

## Abstract

**Objective:** Modern medicine needs to shift from a wait and react, curative discipline to a preventative, interdisciplinary science aiming at providing personalized, systemic, and precise treatment plans to patients. To this purpose, we propose a “digital twin” of patients modeling the human body as a whole and providing a panoramic view over individuals' conditions.

**Methods:** We propose a general framework that composes advanced artificial intelligence (AI) approaches and integrates mathematical modeling in order to provide a panoramic view over current and future pathophysiological conditions. Our modular architecture is based on a graph neural network (GNN) forecasting clinically relevant endpoints (such as blood pressure) and a generative adversarial network (GAN) providing a proof of concept of transcriptomic integrability.

**Results:** We tested our digital twin model on two simulated clinical case studies combining information at organ, tissue, and cellular level. We provided a panoramic overview over current and future patient's conditions by monitoring and forecasting clinically relevant endpoints representing the evolution of patient's vital parameters using the GNN model. We showed how to use the GAN to generate multi-tissue expression data for blood and lung to find associations between cytokines conditioned on the expression of genes in the renin–angiotensin pathway. Our approach was to detect inflammatory cytokines, which are known to have effects on blood pressure and have previously been associated with SARS-CoV-2 infection (e.g., CXCR6, XCL1, and others).

**Significance:** The graph representation of a computational patient has potential to solve important technological challenges in integrating multiscale computational modeling with AI. We believe that this work represents a step forward toward next-generation devices for precision and predictive medicine.

## 1. Introduction

Modern medicine is shifting from a wait and react, curative discipline to a preventative, interdisciplinary science aiming at providing personalized, systemic, and precise treatment plans to patients. Systems and network medicine are rapidly emerging in medical research providing new paradigms to address.

In the next decades, precision and predictive medicine will have a pivotal role in revolutionizing the healthcare system making it more flexible and efficient. Precision and predictive medicine are challenging research fields as they need to deal with the complexity of the human body (Ginsburg and Willard, 2009; Naylor and Chen, 2010). Precision requires integrating large amount of observations at individual and population levels simultaneously. These measures need to be taken at different scales, from genome to clinical and family history and at systemic levels, i.e., considering multiple tissues and organs. In the last years, systems and network medicine have introduced a variety of novel approaches with the aim of integrating and gaining knowledge on the human body. We have no capacity to integrate such disparate information into equation-based models but we can use machine learning and, in particular, deep learning methods to achieve this integration goal.

The primary objective of this work is exploring challenges and opportunities in modeling the human body as a whole, providing a panoramic view over individuals' conditions. To this aim, we propose a proof of concept of a “digital twin,” i.e., a virtual prototype of patients mirroring the underlying biological system (Gelernter, 1993; Laubenbacher et al., 2021) combining information at organ, tissue, and cellular level. Existing prominent examples of digital twins in healthcare include “the artificial pancreas” (Brown et al., 2019; Kovatchev, 2019), pediatric cardiac digital twins (Gutierrez et al., 2019; Shang et al., 2019), and diabetes models (Eddy and Schlessinger, 2003). However, all these examples focus on just one single aspect of the human body due to its extreme complexity. As a result, they are not suitable to provide a holistic overview over the whole human body. We believe that recent graph representation approaches could overcome digital twin's limitations scaling across all the variety of body signals at different levels, making possible a revolution in healthcare. This work provides a first proof of concept providing the first elements for a novel class of machine-learning-assisted tools that scale to medical device deployment and run time monitoring and verification. By fusing ideas from systems medicine with scientific computing and machine learning, our software integrates and automates the analysis of vital parameters models under large uncertainty. A high degree of automation could transform how we use models in the scientific and medical discovery cycle and open up for a next-generation of powerful medical devices for probing the inner workings of full body in well-being and disease conditions.

**Figure 1:**
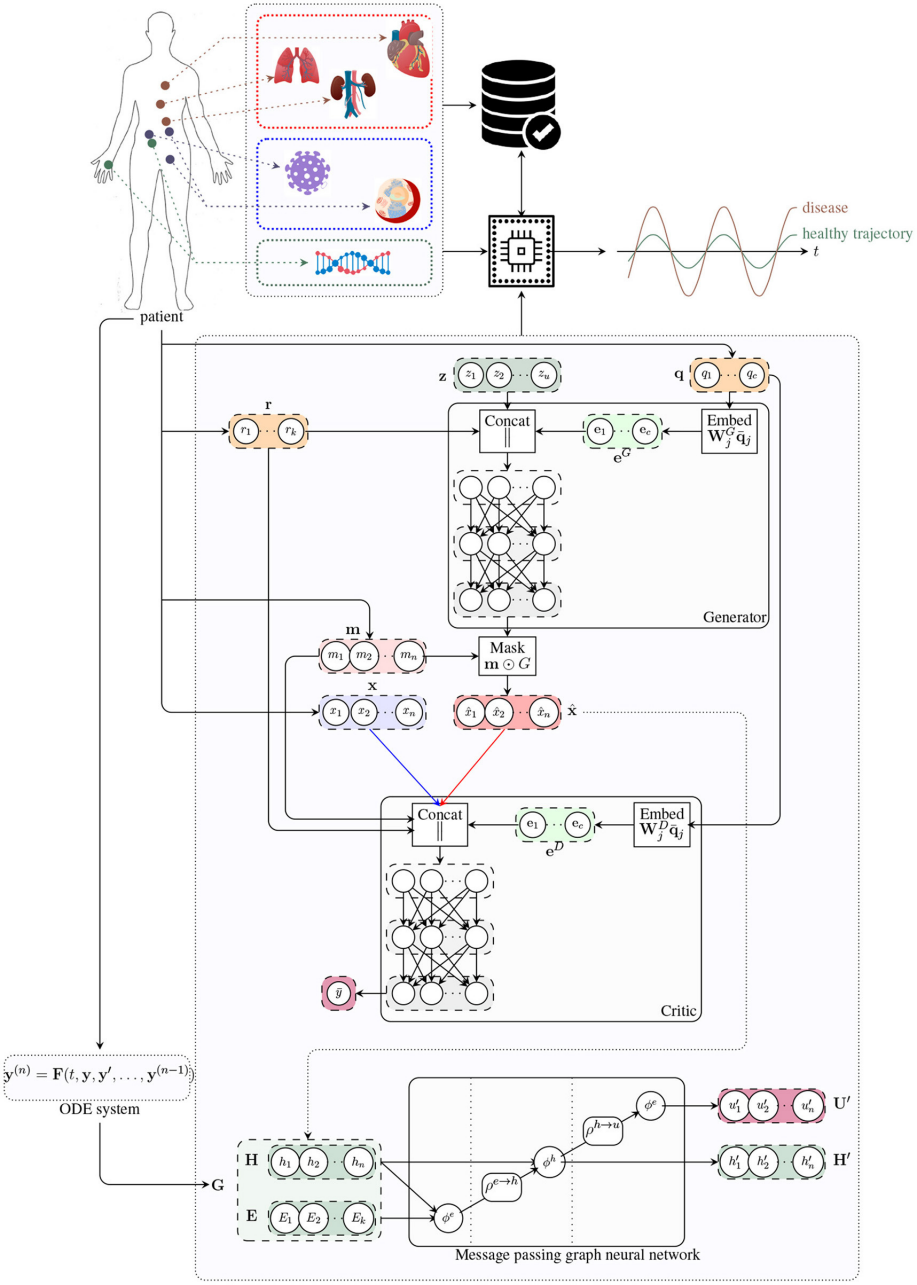
Architecture of the digital twin model. The generator receives a noise vector z, and categorical (e.g. tissue type; q) and numerical (e.g. age; r) covariates, and outputs a vector of synthetic data (x^). The critic receives data from two input streams (real, blue; and synthetic, red), a mask m indicating which components of the input vector are missing, and the numerical r and categorical q covariates. The critic produces an unbounded scalar $\bar{y}$ that quantifies the degree of realism of the input samples from the two input streams. The handcrafted ODE system proposed in Barbiero and Lió (2020) is used to determine a graph representation of patient's physiology. The message passing neural network updates latent node features to estimate global attributes describing the evolution of the underlying physiological system.

The proposed architecture combines the qualities of generative and (Goodfellow et al., 2014) graph-based models (Scarselli et al., 2008) (see Figure 1). On the one hand, the generative model can be used to produce synthetic data under different biological states that might not be observed in reality. By augmenting the set of explorable states of the underlying biological system, the generative model may be employed for the simulation of extremely rare clinical scenarios representing precarious conditions, which might be difficult to analyze otherwise (Yi et al., 2019). In clinical contexts, this means that physicians will be able to set up personalized experiments in a virtual environment representing their patients in a very detailed and realistic way. On the other hand, the graph model represents the actual digital twin, providing a general and flexible framework to run probabilistic simulations. A panoramic view of individuals' conditions is provided by the final network configuration that combines information at organ, tissue, and cellular level. Cross-modal signals are also supported by the most recent graph learning frameworks, thus allowing the combination of different data sources, both structured and unstructured, real or simulated by generative methods. Finally, by relying upon flexible and modular architectures, our “digital twin” model can be conveniently deployed in dedicated hardware modules paving the way for a next-generation of medical devices.

## 2. Design of a Biomedical Digital Twin

The birth of the term “digital twin” could be the NASA's Apollo program where one spacecraft was launched into the outer space, while a “twin” spacecraft remained on earth to mirror flight conditions. Digital twin has been defined as “an integrated multiphysics, multiscale, probabilistic simulation of a vehicle or system that uses the best available physical models, sensor updates, fleet history, etc., to mirror the life of its flying twin” (Shafto et al., 2010; Grieves, 2015). The digita l twin is a virtual prototype; the analysis of its digital life cycle provides information to understand a product's functionality, manufacturing, behavior, and usage prior to building it. Here, the meaning of digital twin is slightly different: there is no product to be built, instead experimenting therapies on a digital twin will be cost-effective and will provide us with a rigorous testbed to conduct medical interventions. Within this framework, the artificial intelligence model could enable the prediction of disease trajectories before the insurgence of symptoms. The personal medical digital twin could also represent a pragmatic way for the cyber-physical fusion, as a new approach to support biomedical engineering design. In our vision, a composable AI architecture could enable the development of automatic analysis and verification techniques that are key to translational medicine.

**Figure 2:**
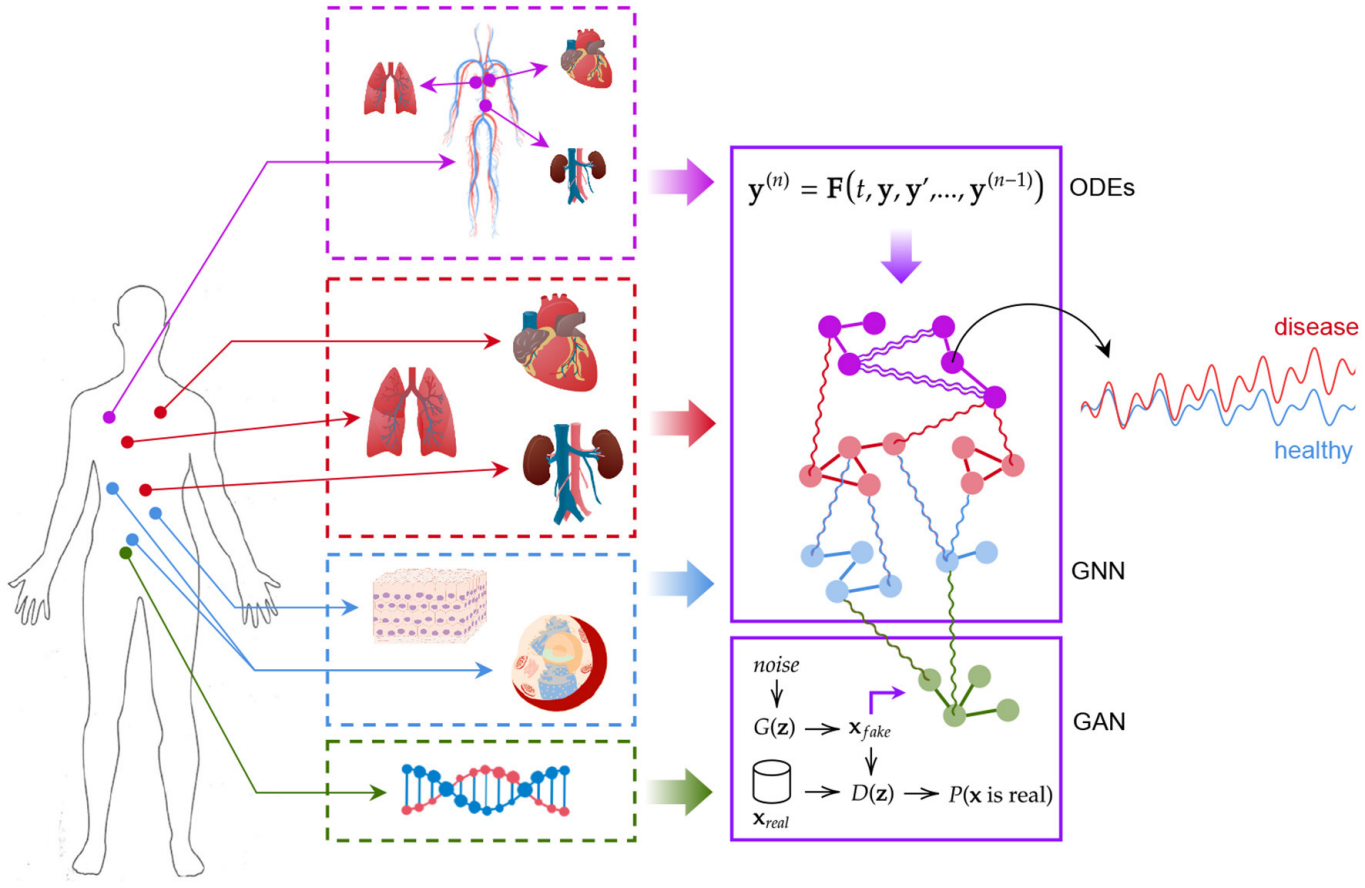
The digital twin model. Ordinary differential equations, graph neural networks, and generative adversarial networks are used synergically to model patient's conditions.

Our digital twin consists of a modular AI-aided system that can be used to model the human body as a whole and to forecast the evolution of pathophysiological conditions (see Figure 2). The first module is based on a graph neural network (GNN) forecasting clinically relevant endpoints (such as blood pressure), while the second one is represented by a generative adversarial network (GAN) providing a proof of concept of multi-omic integrability.

### 2.1. The Effectiveness of GNNs and GANs in Biomedical Signal Analysis

The lack of interpretability of deep learning models has been one of the most significant barriers preventing their application in healthcare. Such models exhibit great capacity (Hornik, 1991) but understanding their behavior and following their decision-making process is not trivial (Castelvecchi, 2016). There is a growing body of literature focusing on interpretable artificial intelligence and interpretable deep learning aiming at developing white box models or at explaining black box ones (Das and Rad, 2020). Among such techniques, GNNs have started drawing the attention of both research and industry communities (Bronstein et al., 2017; Zhou et al., 2018). Such models are much more interpretable with respect to other neural approaches thanks to their graph structure, which is quite easy to understand from a human standpoint, and a few studies have already shown how graph networks can be effectively employed in biology and healthcare (Zitnik et al., 2018; Gysi et al., 2020).

Several properties of graph and generative adversarial neural networks make them suitable for medical data analysis. *(1) Non-linearity*: Both GNNs and GANs are able to detect non-linear patterns, which is of key interest as most systems are inherently non-linear in nature. Examples in medicine include heart rate dynamics, pulmonary functions, vascular structure, and gait dynamics. There is often a loss of non-linearity and multiscale fractal in aging and disease conditions (Goldberger et al., 2002). *(2) Interpretability*: Graph-based models are much easier to interpret with respect to other neural approaches thanks to their structure. The possibility of interpreting the behavior of models and the reason for their predictions is pivotal if not critical in many fields including clinical practice. *(3) Non-Euclidean geometry*: As a unique non-Euclidean data structure for machine learning, graphs can be used to model a variety of biological systems at different scales. Tissue and organ distributions could be modeled as graph models where each node or the graph contain time-dependent signals, similarly for pressure and electric sensors positioned at various parts of the body. Lymphatic vessels can also be modeled as a network where lymph nodes are vertices. At lower scale, cell arrangements in tissues form particular manifolds; proteins and genes are organized in regulatory networks; other examples are cytoskeleton and organelles (mitochondria networks). Additionally, diseases could be seen as nodes in a graph where edges represent comorbidity or underlying polygenic causes. *(4) Modularity*: A key property of GNNs is modularity, which allows to learn independent mechanisms that can be reused in several parts of the graph. Modularity facilitates scalability and allows to model dynamic properties of graphs. *(5) Cross-modality*: Both GNNs and GANs can learn how to combine structured and unstructured data sources, spanning different levels of biological complexity. This is particularly relevant when integrating signals at different levels of biological scale such as DNA methylation and functional magnetic resonance imaging (fMRI) data. *(6) Generative*: Both GNNs and GANs can learn how to generate new data preserving the statistical properties of the training set. This could be used to compare statistics at individual level with those at specific groups identified with stratification analysis or at general population levels. *(7) Multiscale*: The graph representation has the capability of integrating granular information organized as networks at different layers of biological complexity. This allows to recognize patterns in higher-order structures such as motifs, pathways, tissues (as compositions of cells), organs (as composition of tissues), processes and apparatus (as composition of organs), and stratification (as composition of individuals). *(8) Spectral density*: Together with spatial properties, GNN are amenable to frequency domain analysis. This allows to investigate network motifs, substructures, and periodical patterns at network levels.

### 2.2. Graph Neural Model

Graphs are mathematical structures that are used to model a set of objects (nodes) and their mutual relationships (edges) (Bollobás, 2013). Graphs are employed in a variety of research areas as they provide a general and flexible data structure for modeling real-world systems (Lieberman et al., 2005; Zhou et al., 2018; Rakocevic et al., 2019; Bica et al., 2020). GNNs are deep learning-based models working on the graph domain (Scarselli et al., 2008; Battaglia et al., 2018; Wu et al., 2020). Their properties have been recently drawn the attention of the artificial intelligence research community given their high interpretability (Lecue, 2019; Huang et al., 2020). The combination of graph theory and neural network elements have made GNNs one of the most promising tools to analyze complex systems in the graph domain. From neural networks, GNNs inherit a data-driven approach associated with a multi-layer architecture, which is the key to extract hierarchical patterns from data. However, unlike other deep-learning models, GNNs exploit additional features from graph theory and other mathematical disciplines. The main advantage with respect to other machine learning models relies in their extremely flexible and interpretable architecture. Once defined, the main endpoints of a system together with their mutual relationships directly induce a corresponding graph representation, which can be easily interpreted from a human standpoint. The abstract graph representation can be handcrafted, when the complexity of the underlying system allows it, or even automatically induced from data using generative approaches (Li et al., 2018). Hybrid techniques may also be explored taking advantage of generative algorithms for handling system complexity and human modeling to customize the most relevant endpoints. The design of GNNs is based on two basic principles, flexibility, and composability. GNNs support different graph structures as well as flexible representations of global, node, and edge attributes, customizable according to specific demands of tasks.

#### 2.2.1. Stratification of Human Body Layers in a GNN

GNNs natively allow the design of complex systems using a modular approach. First, the complexity of the human body is broken up by developing independent subsystems representing genomic alterations, biological pathways, and organ physiology. Each subsystem can be represented as a different node or a network of nodes in a GNN, while inter-process signals can be reframed as message passing operations supporting multiscale ripple effects. Homogeneous subsystems can be aggregated into layers according to their characteristics. Our digital patient model is composed of four biological layers: the transcriptomic layer, the cellular layer, the organ layer, and the exposomic layer. Other layers can be easily implemented.

##### 2.2.1.1. Transcriptomic Layer

The transcriptomic layer operates on the set of RNA transcripts produced by the genome at a particular time. Currently, RNA sequencing (RNA-seq) can measure RNA abundance across the entire genome with high resolution. The resulting high-throughput gene expression data can be used to uncover disease mechanisms (Emilsson et al., 2008; Cookson et al., 2009; Gamazon et al., 2018), propose novel drug targets (Evans and Relling, 2004; Sirota et al., 2011), provide a basis for comparative genomics (Colbran et al., 2019), and address a wide range of fundamental biological problems.

In this work, we study the crosstalk between tissues in the organ layer (see Figure 1) through the communicome, e.g., communication factors in blood (Ray et al., 2007). Specifically, we analyze to what extent the expression of genes involved in the renin–angiotensin system (RAS) can be explained by genes from signaling and receptor pathways, including the chemokine, TNF, and TGF-β pathways. We further develop a transcriptomics generative model based on a generative adversarial network (Goodfellow et al., 2014) and simulate the effects of SARS-CoV-2 infection by conditioning on high expression of ACE2 in the lung, kidney, and pancreas.

##### 2.2.1.2. Cellular Layer

The cellular layer involves biological processes affecting individual cells from metabolism and protein synthesis to replication and motility. In this study, we focus on modeling the RAS, one of the main biological pathways regulating blood pressure and closely related to SARS-CoV-2 infectivity. Hence, it represents a suitable case study to demonstrate the flexibility and expressiveness of our GNN-based approach. The RAS is a hormone system regulating vasoconstriction and inflammatory response (Fountain and Lappin, 2019). The key hormone of the system is the peptide angiotensin II (ANG-II) generated from the decapeptide angiotensin I by the angiotensin-converting enzyme (ACE). ANG II promotes vasoconstriction, hypertension, inflammation, and fibrosis by activating the ANG-II type 1 receptor (AT1R) (Kuba et al., 2010; Gironacci et al., 2011). Glucose concentration, ACE inhibitor treatments, and viral infections binding to ACE2, such as SARS-CoV-2, can all have a significant impact on the RAS. A high glucose concentration may determine chronic hypertensive conditions. Reducing ANG II production with ACE inhibitors increases vasodilation and vasoprotection effects stimulated by the overproduction of AT2R and ANG-(1-7) (Zaman et al., 2002). Viral infections such as SARS-CoV-2 may also have an impact on RAS, as the virus binds to ACE2 in order to gain entry into the host cell. This results in an altered ACE2 activity and concentration, possibly leading to hypertension and inflammatory response (South et al., 2020).

##### 2.2.1.3. Organ Layer

The organ layer comprises group of tissues with similar functions (organs) and complex networks of cooperating organs. Given the nature of the multi-factorial disease under study, we limited the organ layer to the circulatory system and a physiological representation of a few organs (Barbiero and Lió, 2020): heart, lungs, and kidneys. The heart model includes four compartments known as chambers (Neal and Bassingthwaighte, 2007). Deoxygenated blood collected from the superior and inferior venae cavae flows into the right atrium. When the right atrium contracts, the blood is pumped through the tricuspid valve into the right ventricle. From the right ventricle, the blood is pumped into the pulmonary trunk through the pulmonary valve flowing toward the lungs where carbon dioxide is exchanged for oxygen. The pulmonary circulation is composed of five vascular segments: proximal and distal pulmonary artery, small arteries, capillaries, and veins. Oxygenated blood collects into the left atrium via the pulmonary veins. From there, it flows into the left ventricle through the mitral valve and it is pumped into the aorta through the aortic valve for systemic circulation, providing oxygen and nutrients to body cells for metabolism in exchange for carbon dioxide and waste products. The mean arterial blood pressure is controlled by baroreceptors, special sensory neurons excited by a stretch in the carotid sinus and aortic arch vessels. They relay sensory information regarding blood pressure changes to the central nervous system where it is processed and utilized primarily in autonomic reflexes, regulating short-term blood pressure.

##### 2.2.1.4. Exposomic Layer

The exposome refers to the totality of exposure individuals experience from conception until death and its impact on chronic and acute diseases (Wild, 2005). Toxicants, dietary regimens, treatments, physical exercise, posture, and lifestyle habits are possible exposures taking part to individual's well-being or disease condition. All such environmental factors are deeply coupled among themselves but also with individuals influencing the effects of new or present exposures. The exposome is intrinsically co-dependent on a person's genetics, epigenetics, health status, and physiology. For instance, regular exposure to pollution may lead to the outbreak of a lung carcinoma, which in turn may call for clinical intervention. In this work, we consider four types of exposures: dietary habits, physical activity, therapeutic treatments, and viral infections.

#### 2.2.2. Inter-process Signals and Clinical Endpoints

One of the main advantages of using GNN-based models relies in that inter-process and multiscale communications can be natively implemented using message passing. In a GNN, each biological entity can be represented as a node, while the relationship between two entities can be modeled using directional edges. Signals exchanged between nodes are implemented using message functions ϕ^*h*^ (see Equation 1), which are used to update the hidden states of nodes. Such state transition will then have an impact on messages exchanged at the following time steps. Another strength of GNN models consists of the possibility of supervising the evolution of the underlying system by using the readout functions ϕ^*u*^. Hence, the endpoints of multi-factorial diseases can be directly controlled by checking the output of readout functions in critical nodes. The resulting GNN model will combine a simple and modular design with a versatile structure accommodating for complex multiscale systems where clinical endpoints can be easily monitored and forecast in real time.

### 2.3. Generative Adversarial Model

**Figure 3:**
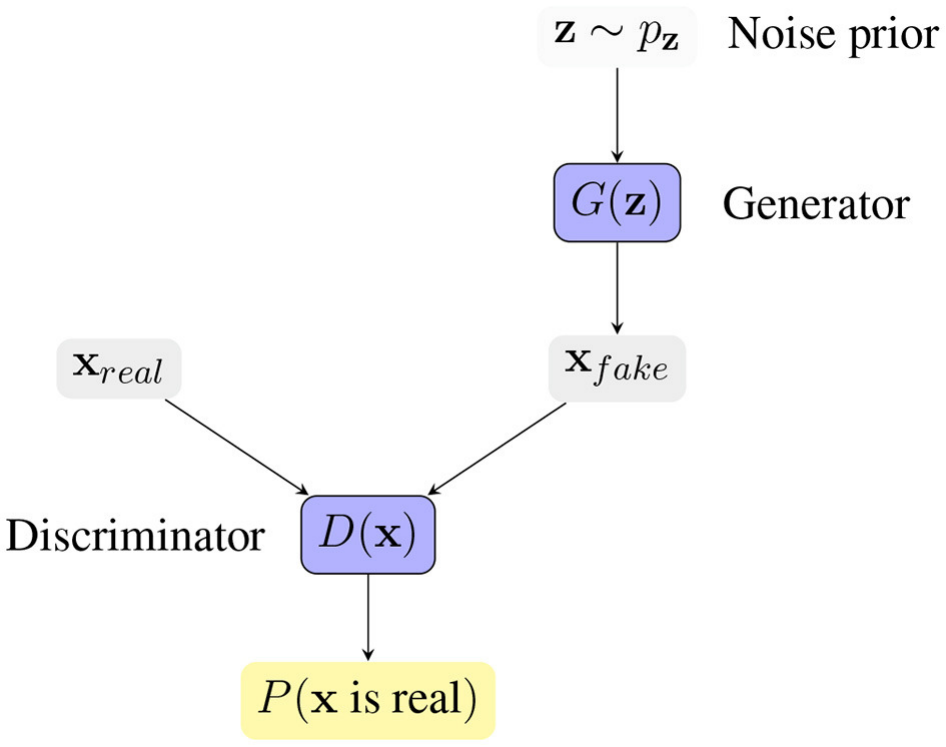
Generative Adversarial Network framework. The generator *G*(z) receives a vector z sampled from a noise prior distribution *p*_z_, and generates a synthetic sample *x*_*f*_*ake*. The discriminator *D*(x) tries to distinguish *real* samples from *fake* samples, producing the probability of x coming from the *real* data distribution. The competition between the two players drives the game and makes both players increasingly better.

One way of studying probability distributions is by means of generative models, which describe the random phenomenon in terms of the joint probability distribution of observed and target variables (Jebara, 2012). Generative adversarial networks (GANs) are a framework for estimating generative models via an adversarial process (Goodfellow et al., 2014). They are often described as a two-player game in which both players are encouraged to improve. One player, the *generator*, creates samples that are intended to be indistinguishable from the ones coming from a given data distribution. The other player, the *discriminator*, learns to determine whether samples come from the *fake* distribution (*fake* samples) or the *real* data distribution (*real* samples). Figure 3 shows the basic idea of generative adversarial networks. With respect to other generative models, they provide a general and flexible framework for the analysis of joint probability distributions. The architecture itself allows a fine control of the data generation process and a high level of customization, making them suitable for a variety of experimental scenarios.

#### 2.3.1. Crosstalk Between Tissue Types

The activity of biological systems is determined by internal factors, determined by intrinsic and functional properties, and by external factors shaping the interconnections between different systems. Chemical and molecular events, like oxygenation or protein phosphorylation, are often the vehicles of biological signals' transduction. A chain of biochemical events forms a signaling pathway whose activation may give rise to a biochemical cascade of events affecting the organism at different levels. In complex organisms, several signal transduction pathways communicate and react reciprocally generating biological crosstalks. Crosstalks have been widely characterized and observed in a variety of biological processes from micro- to macroscale from genomics (Poyton and McEwen, 1996; Du et al., 2015), to internal and external cell activity (Geiger et al., 2001; Li et al., 2016), and even between tissues (Lengyel et al., 2018). In particular, receptors and signaling factors from the chemokine, TNF, and TGF−β pathways are known to take an active role in tissue communication as well as inflammatory-associated diseases (e.g., cardiovascular diseases affecting that the heart and the stiffness of blood vessels). Here, we develop a generative model based on a generative adversarial network to produce synthetic transcriptomics data describing the ripple effects of a viral infection on crosstalks between different tissues. The aim is to demonstrate how generative approaches can be used both to reproduce and enhance the set of observable states of a patient allowing for a deeper understanding of complex biological processes.

## 3. Results

### 3.1. Clinical Case Studies

In Barbiero and Lió (2020), the authors proposed a computational tool for running simulations integrating a variety of mechanistic and phenomenological models describing the human body with ordinary differential equations (ODEs). This computational framework is hereby used to generate two clinical case studies. The main difference of the proposed approach with respect to the computational tool proposed in Barbiero and Lió (2020) consists of a different modeling approach based on state-of-the-art AI models instead of ODEs.

The first scenario consists of an elderly patient experiencing hypertension and type 2 diabetes with diabetic nephropathy. Her lifestyle is mainly sedentary and her diet is rich in carbohydrates. The patient needs a therapeutic plan for the treatment of her hypertension. The task for the clinician is to personalize the therapy assigning a proper daily dosage of benazepril. This case study is used to show how the digital patient model can be employed to simulate the evolution over time of clinical endpoints under a set of possible therapeutic plans and to choose the best option.

In the second scenario, the same patient is seeking medical help for a mild flu caused by a SARS-CoV infection. For this case study, the model can be used to constantly monitor and forecast clinical endpoints to prevent complications threatening patient's life. The decreased oxygenation caused by flu may have detrimental effects on both heart and brain activities indeed. Studies have reported that SARS-CoV infections can activate the blood clotting pathway by impairing left heart pumping performance, which results in a blood back up in the lungs and in a increased blood pressure. High blood pressure can reduce blood vessel's compliance decreasing blood and oxygen flows and leading to a higher risk of developing systemic conditions. For this reason, heparin-based therapies have been recommended to prevent clot formation or tissue plasminogen activator (tPA) (Sardu et al., 2020; Tang et al., 2020). Although some variation in blood pressure throughout the day is normal, a high blood pressure variability is associated with a higher risk of cardiovascular disease (O'Rourke and Nichols, 2005; Mitchell et al., 2010; Wen et al., 2015; Clark et al., 2019; Bangalore et al., 2020) and all-cause mortality (Tao et al., 2017; Kim et al., 2018). Clogged arteries, fibrosis, and strokes caused by blood pressure spikes are among the main complications threatening patient's life and calling for the foremost necessity for treatment. Hence, blood pressure is one of the most relevant clinical endpoints that need to be constantly monitored in real time and accurately forecast.

### 3.2. Forecasting Clinical Scenarios

#### 3.2.1. Dataset

Our digital twin model is hereby used to actively monitor and forecast the endpoints highlighted in the two clinical case studies. First, the computational system described in Barbiero and Lió (2020) based on ordinary differential equations (ODEs) is used to generate a time series of clinical endpoints for each differential equation with a window size of τ = 500 time steps (Barbiero and Lio, 2020). Time series are collected, randomly shuffled, and stacked in a dataset. Each item of the collection is randomly assigned either to a training (*n*_*train*_ = 3, 200), validation (*n*_*val*_ = 800), or test set (*n*_*test*_ = 1, 000).

#### 3.2.2. Training

**Figure 4:**
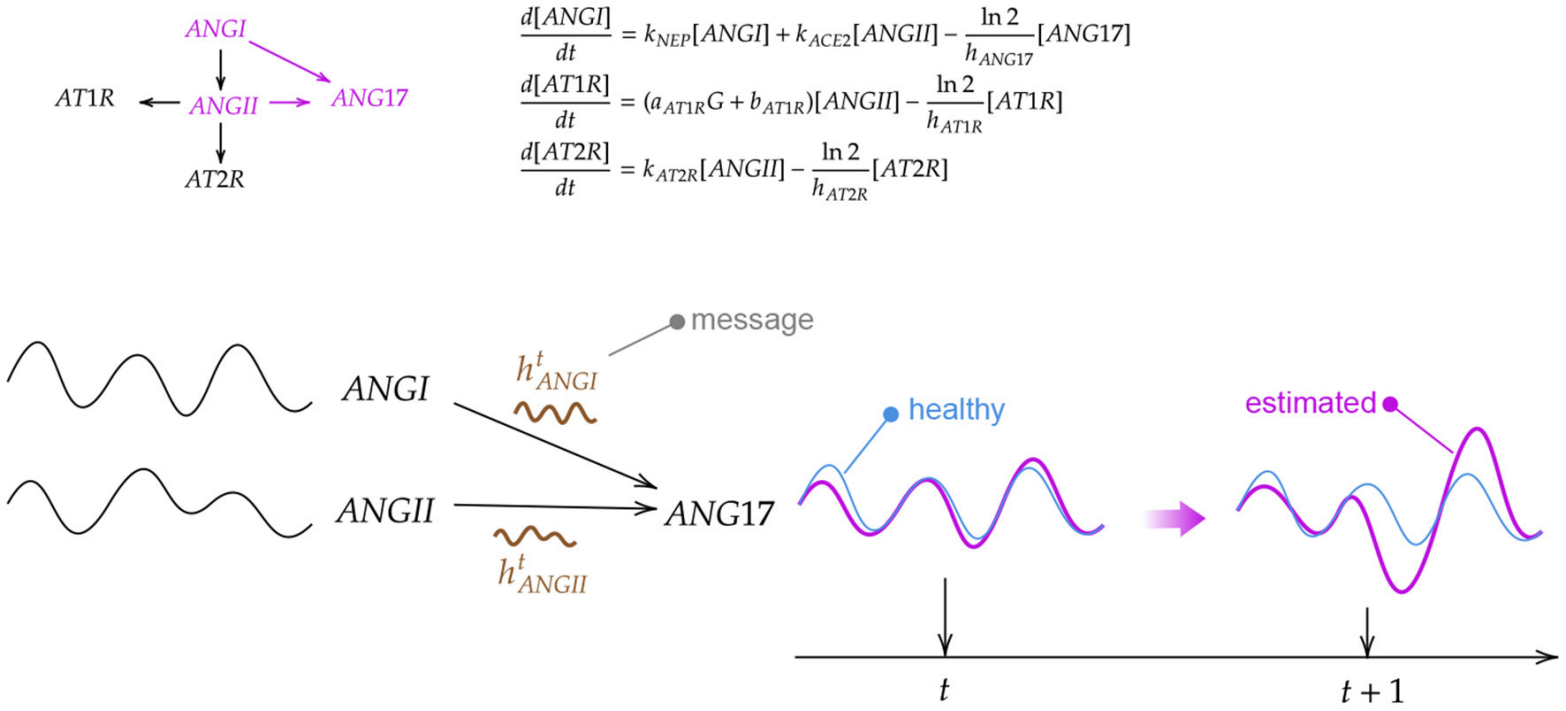
Example of how a biological system can be modeled in a graph neural network through differential equations. First, an ordinary differential equation (ODE) system is derived from a biochemical reaction network. Then, the ODE system is solved for different initial conditions generating a set of trajectories for each variable. Finally, a graph neural network aggregates the information coming from neighbor nodes to update the current state of the variable.

The graph model is derived from the structure of the ODE system, thus leveraging human knowledge (an example is shown in Figure 4). Nodes correspond to variables represented by the differential equations in Barbiero and Lió (2020) while edges follow the underlying relationships. In a GNN-based model, each node learns a latent representation of the state using the messages received from its neighborhood. Hence, the rigid mathematical structure of the ODE system is relaxed in our model as such structure can be learned directly from data. The learning process lasts for η = 50 epochs with a learning rate of ϵ = 0.01. Once trained and validated, the model is used to generate a bundle of possible trajectories for the elements of the test set. As a result, the model estimates a 95% confidence interval of the evolution of each variable over time.

#### 3.2.3. Results

Providing a complete overview of the clinical state of a patient is not trivial. Focusing just on one endpoint might be misleading. On the contrary, a global vision comprising pathophysiological conditions is required in order to provide a clear and effective overview where organs and physiological systems can be monitored as a whole. One of the most effective approaches consists of applying a dimensionality reduction technique (Van Der Maaten et al., 2009) condensing the information of each organ and projecting forecasts in a lower-dimensional space.

**Figure 5:**
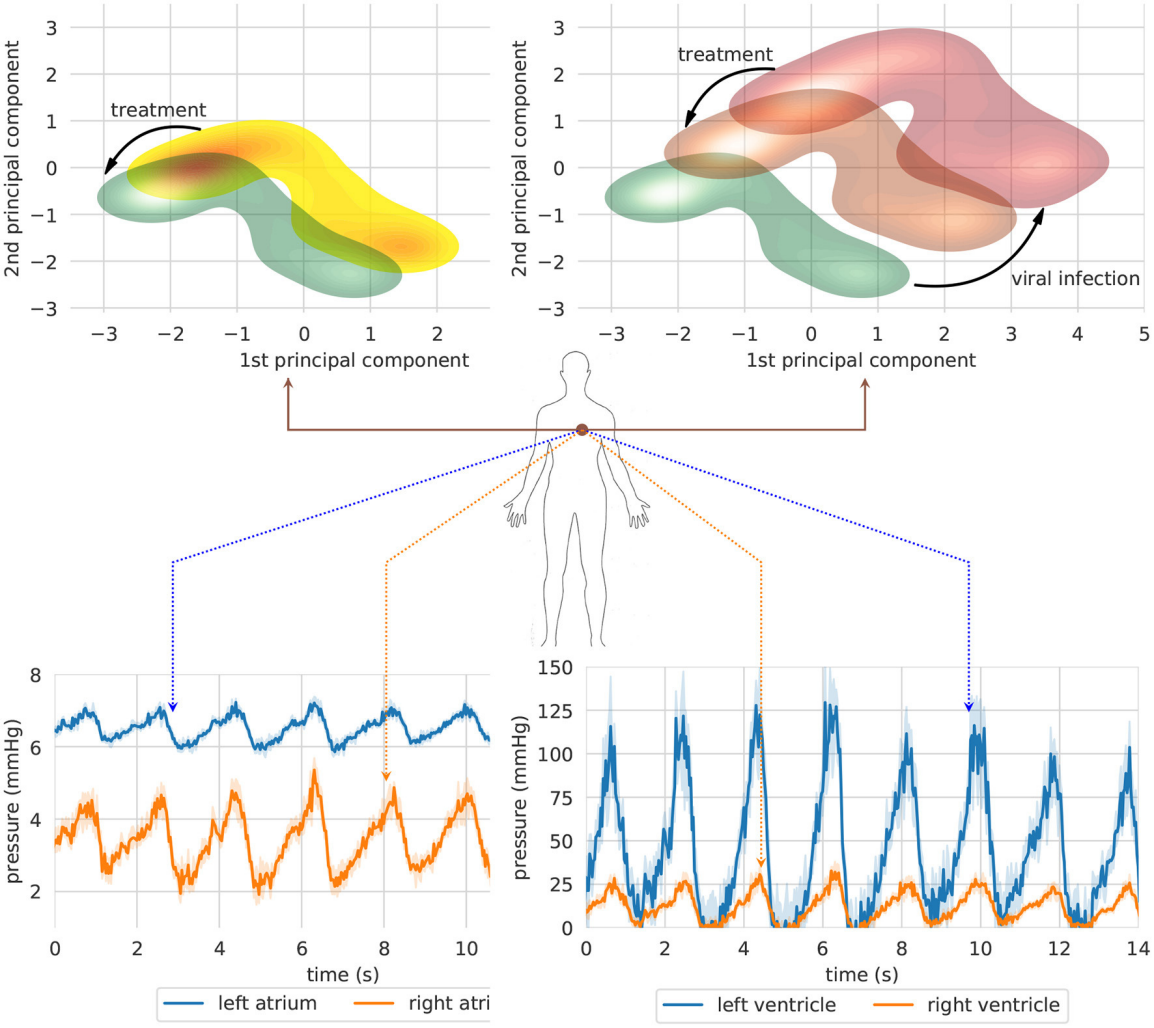
Two clinical case studies represented in a projected heart phase-space. The first case study (left) shows the effect of a therapeutic intervention comprising an increased physical exercise, a reduced amount of calorie intake, and the subscription of a daily dosage of Benazepril (5 mg). The second simulation (right) shows the long-term impact on blood pressure of an untreated SARS-CoV-2 infection (red density) and the effects of a therapy including both Benazepril (5 mg/day) and intra venous injection of heparin (5000 μ/ml) (orange density). (Top) Bundle of predicted trajectories can be visualized and monitored in real time in order to investigate patterns in the time domain. The simulation shows blood pressure in heart chambers starting from healthy state conditions. Error bands represent 95% CI (Bottom).

Figure 5 shows an overview of the clinical state of the heart in a two-dimensional projected phase space. For each clinical case study, a GNN-based model is used to simulate a therapeutic intervention and its impact on blood pressure in heart chambers (right and left atrium and ventricle). In order to provide an overview of heart conditions, we projected the predicted trajectories using principle component analysis (PCA) (Pearson, 1901). The interpretation of both pictures is straightforward. The first one shows the effect of a therapeutic intervention comprising an increased physical exercise, a reduced amount of calorie intake, and the subscription of a daily dosage of benazepril (5 mg). The predicted result of the prescription (green density reporting the 95% CI of the trajectories) reveals an overall reduction of blood pressure mean and variability in heart chambers. This results in a reduced risk of developing severe cardiovascular conditions with detrimental ripple effects for the whole system.

The second figure reports the simulation corresponding to the second case study. The same patient is seeking medical help to treat the first symptoms of a SARS-CoV-2 infection. The first simulation (red density) shows the long-term impact on heart blood pressure of an untreated viral infection. In this case, blood pressure spikes may cause irreparable damages to blood vessel walls, reducing their compliance, and impairing their capacity for adaptation to different environmental conditions. A synergic therapy including both benazepril (5 mg/day) and intravenous injection of heparin (5,000 U/ml) may have a beneficial effect on blood pressure mean and variability (orange density). On the one hand, benazapril lowers blood pressure by inhibiting ACE activity in cleaving ANG-I and producing ANG-II, which is the key RAS regulator of blood pressure. On the other hand, heparin is used to prevent and dissolve blood clots (Sardu et al., 2020; Tang et al., 2020). The treatment has an indirect impact on blood pressure by making blood less dense, reducing clotting formation, and lowering inflammation.

A lower-dimensional representation of an organ or system as a whole could be interesting to get a rapid and clear overview of the long-term impact of a disease or a therapeutic intervention. Nonetheless, bundle of predicted trajectories can be visualized and monitored individually in real time when needed in order to investigate patterns in the time domain. Figure 5 shows an example where blood pressure trajectories in heart chambers are predicted in real time starting from a healthy state condition (green density). In some cases, this representation in the time domain might be closer to common clinical approaches, thus providing a more conventional visualization tool for monitoring clinical endpoints in real time.

### 3.3. Transcriptomics Analysis of the Crosstalk Between Tissue Types

We hypothesize that the communication factors in blood might be playing an important role in the development of the SARS-CoV-2 infection by facilitating the spread of the virus in the human body. Here, we study whether the expression of genes involved in the RAS can be explained by genes that take part of the communicome in blood. This analysis might shed light on whether it is sensible to model the crosstalk between tissue types with a GNN where tissue nodes communicate with each other through whole blood.

#### 3.3.1. Dataset

We leverage data from the Genotype-Tissue Expression (GTEx) project (v8), a resource that has generated a comprehensive collection of human transcriptome data in a diverse set of tissues (Aguet et al., 2019). The dataset contains 15,201 RNA-Seq samples collected from 49 tissues of 838 unique donors. We select genes based on expression thresholds of ≥ 0.1 TPM in ≥ 20% of samples and ≥ 6 reads in ≥ 20% of samples. We normalize the read counts between samples using the trimmed mean of M-values (TMM) normalization method (Robinson and Oshlack, 2010) and we inverse normal transform the expression values for each gene. From all the donors, we select those that have gene expression measurements for whole blood, yielding 670 unique individuals. We then match the patients' whole blood samples with the corresponding measurements in lung (418), cortex of kidney (62), pancreas (257), and left ventricle of heart (324). Finally, we use the KEGG pathway database (Kanehisa et al., 2010) to select genes from the RAS (*hsa04614*), chemokine (*hsa04062*), TNF (*hsa04668*), and TGF-β (*hsa04350*) pathways.

#### 3.3.2. Results

**Figure 6:**
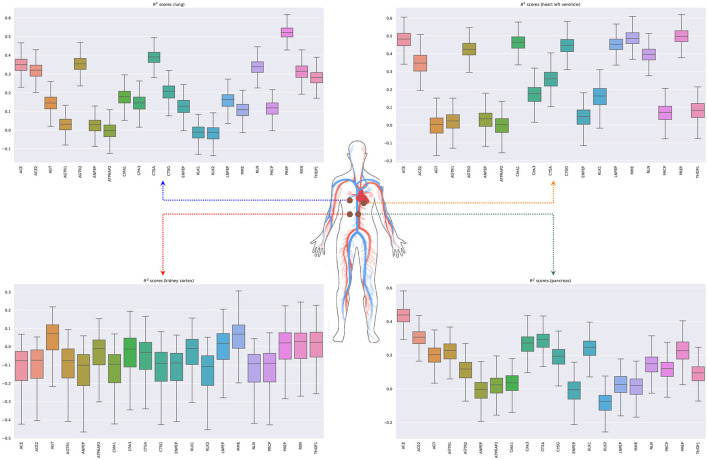
Bootstrapped *R*^2^ scores for genes involved in the renin-angiotensin system for lung, heart (left ventricle), kidney (cortex), and pancreas. The input variables are the expressions of genes in whole blood belonging to the chemokine, TNF, and TGF-β pathways.

Figure 6 shows the bootstrapped *R*^2^ scores for each gene in the RAS pathway in different tissue types. To compute the bootstrapped scores, we sampled donors with replacement (sample size: 75% of the total observations), trained the ridge regression model (Equation 5.3) on the sampled data, and evaluated the performance on the remaining out of bag (OOB) observations. Appendix 3 in Supplementary Material shows the held-out performances for different regularization strengths. We repeated this process 1,000 times to obtain a distribution of *R*^2^ scores for each gene. Our results show that the expression of some genes in the ACE2 pathway can be partially explained by signaling genes from whole blood. Notably, the associations for the kidney (cortex) are weaker or non-existent, potentially because the data are limited for this tissue (62 samples) or because the biological associations are indeed small. Overall, these results suggest that signaling pathways such as TNF, TGF-β, and chemokine might be playing an important role in the development of the SARS-CoV-2 infection.

**Figure 7:**
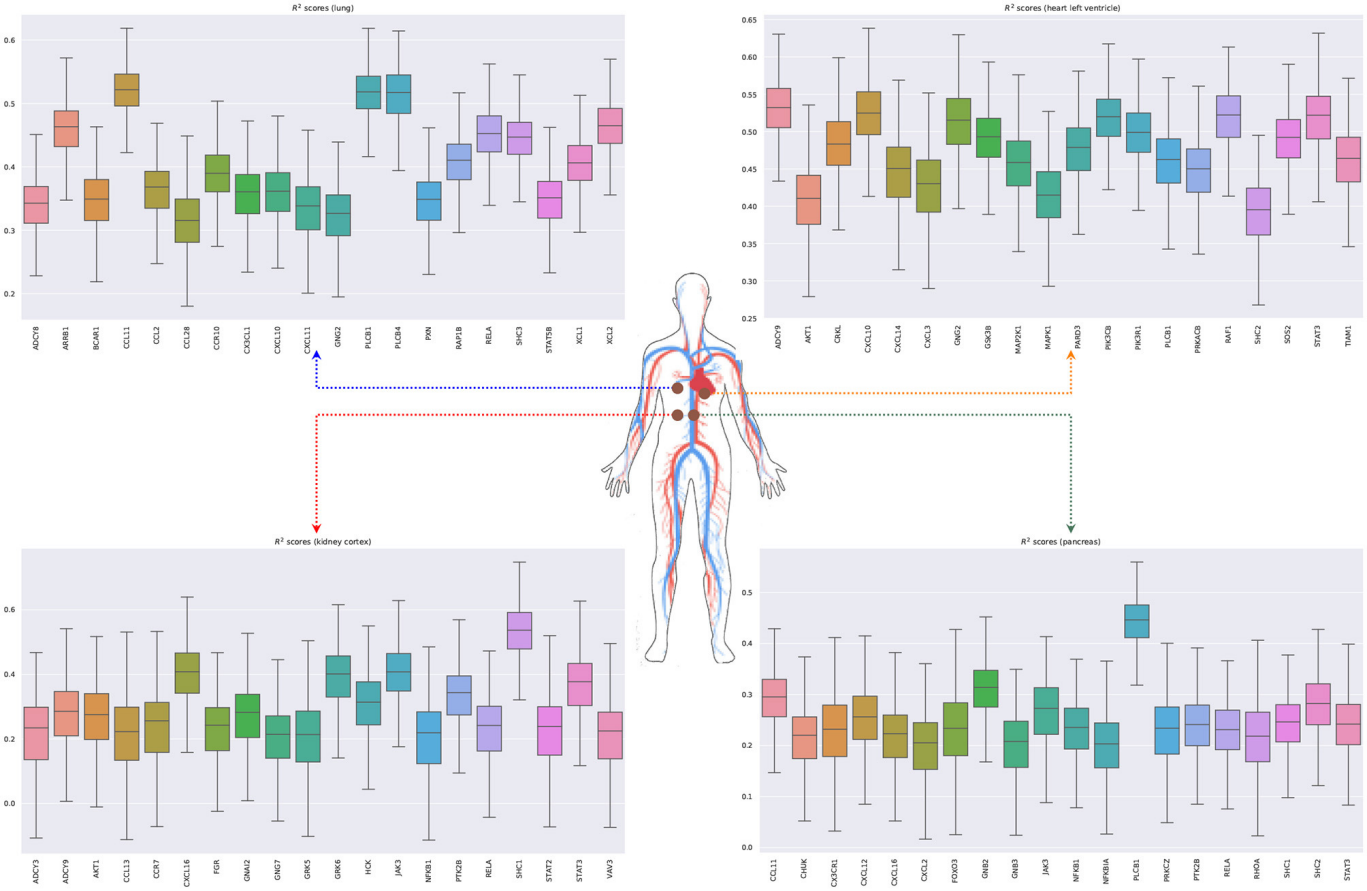
Bootstrapped *R*^2^ scores for several cytokines and receptors for lung, heart (left ventricle), kidney (cortex), and pancreas. For each tissue type, we show the top 20 predicted cytokines. The input variables are the expressions of genes in whole blood belonging to the chemokine, TNF, and TGF-β pathways.

We next model the expression of cytokines and receptors from whole blood (TNF, TGF-β, and chemokine pathways) as a function of cytokines from other tissue types (lung, kidney, heart, and pancreas) (Lijnen et al., 2003; Elmarakby et al., 2007; Rudemiller and Crowley, 2017). Figure 7 shows the bootstrapped *R*^2^ scores for the top 20 cytokines (chemokine pathway) for each target tissue type. These results illustrate the associations between cytokines in blood and other tissue types, which facilitate tissue communication and crosstalks.

### 3.4. Generative Model for Transcriptomics Data

The generative model is here used to produce synthetic transcriptomics data. By conditioning on high expression of ACE2 in the lung, kidney, and pancreas, we aim to simulate the effects of SARS-CoV-2 infection in the expression of genes involved in communicome and signaling pathways such as TNF, TGF-β, and chemokines. These pathways are implicated in many physiological and pathological processes including the regulation of blood pressure and inflammatory processes, and have been hypothesized to play a central role in SARS-CoV-2 infection (Garvin et al., 2020). For this analysis, we use data from the GTEx project previously described. In Appendix 2 in Supplementary Material, we analyze the held-out performance for different architectures of the generator and critic and describe all the training details.

**Figure 8:**
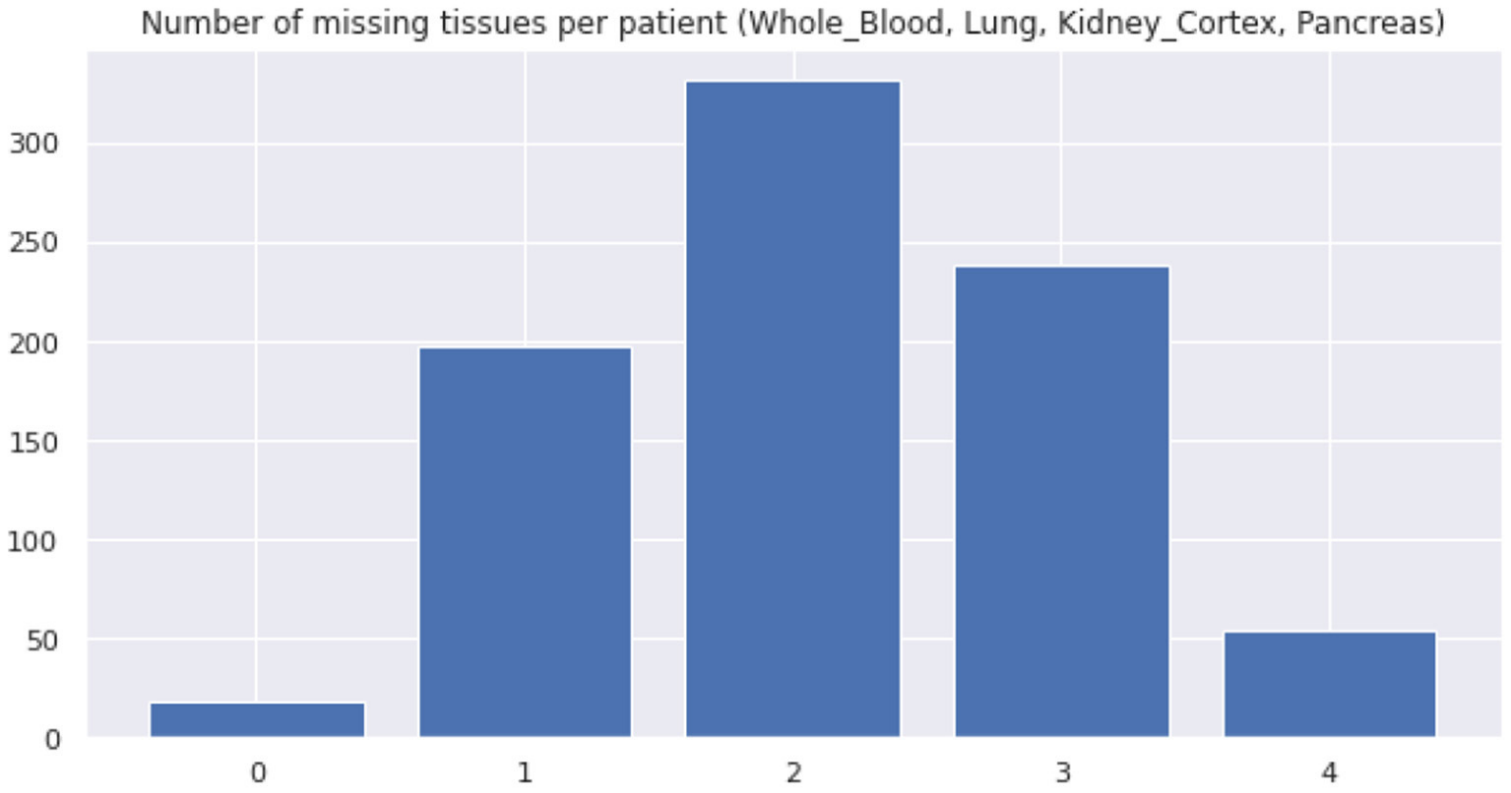
Distribution of missing tissues per GTEx patient. This plot only considers 4 tissue types (whole blood, lung, kidney (cortex), and pancreas).

Real datasets often lack transcriptomic measurements that account for multiple tissue types jointly. For example, out of 838 GTEx donors, only 257 of them present joint observations for pancreas and whole blood (Figure 8 shows the distribution of missing tissues per patient). Importantly, our model allows to sample gene expression data for synthetic patients in every modeled tissue type and without any missing values, facilitating the cross-tissue analysis of gene expression.

#### 3.4.1. Results

**Figure 9:**
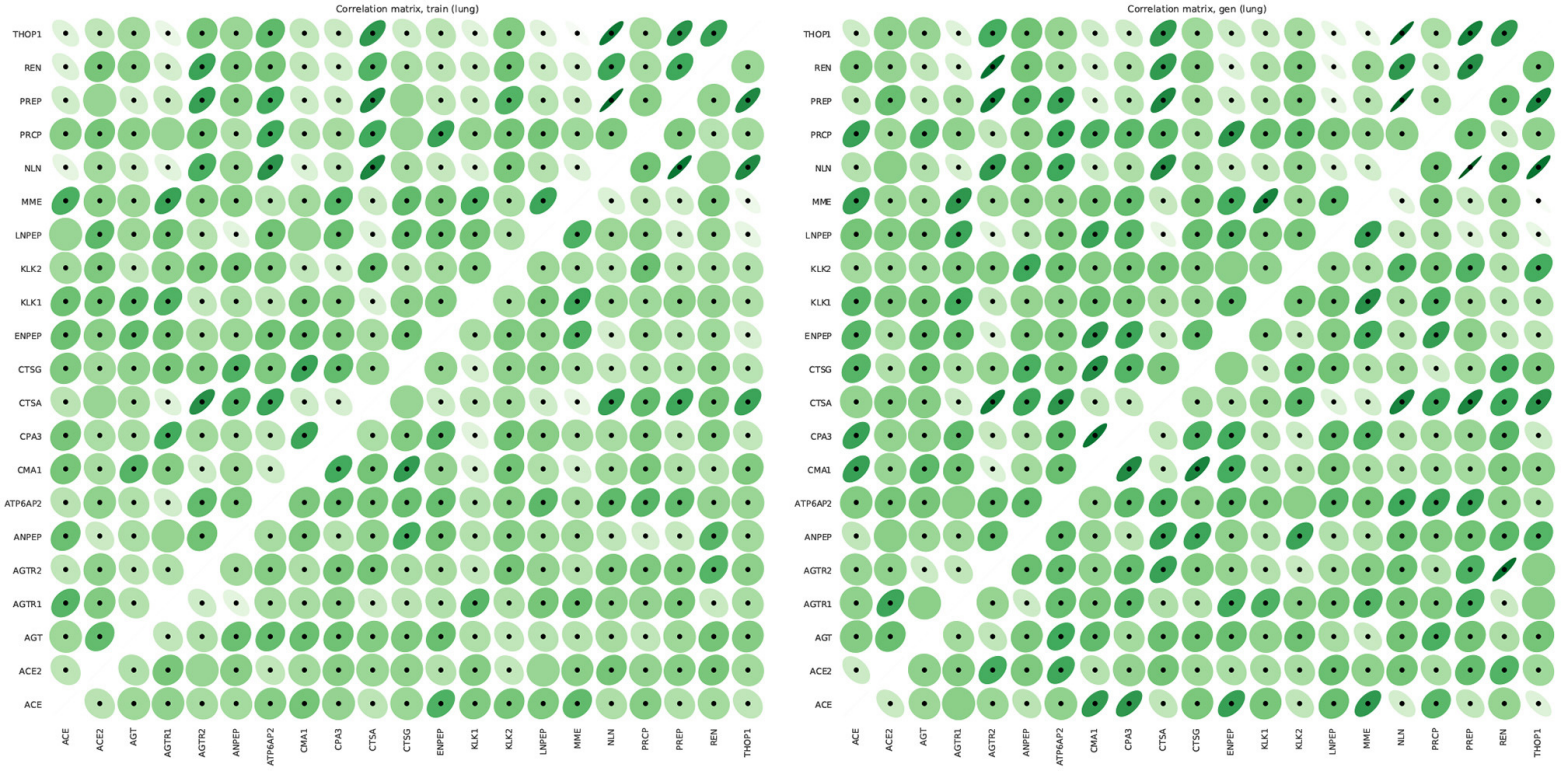
Pairwise Pearson correlations between genes in the renin-angiotensin system pathway in lung for real **(left)** and synthetic **(right)** data. The correlations in the lower and upper matrices are computed from samples with low (61 samples) and high (60 samples) ACE2 expression, respectively. We use dots to label statistically significant correlations (two-sided *p*-value < 0.05).

**Figure 10:**
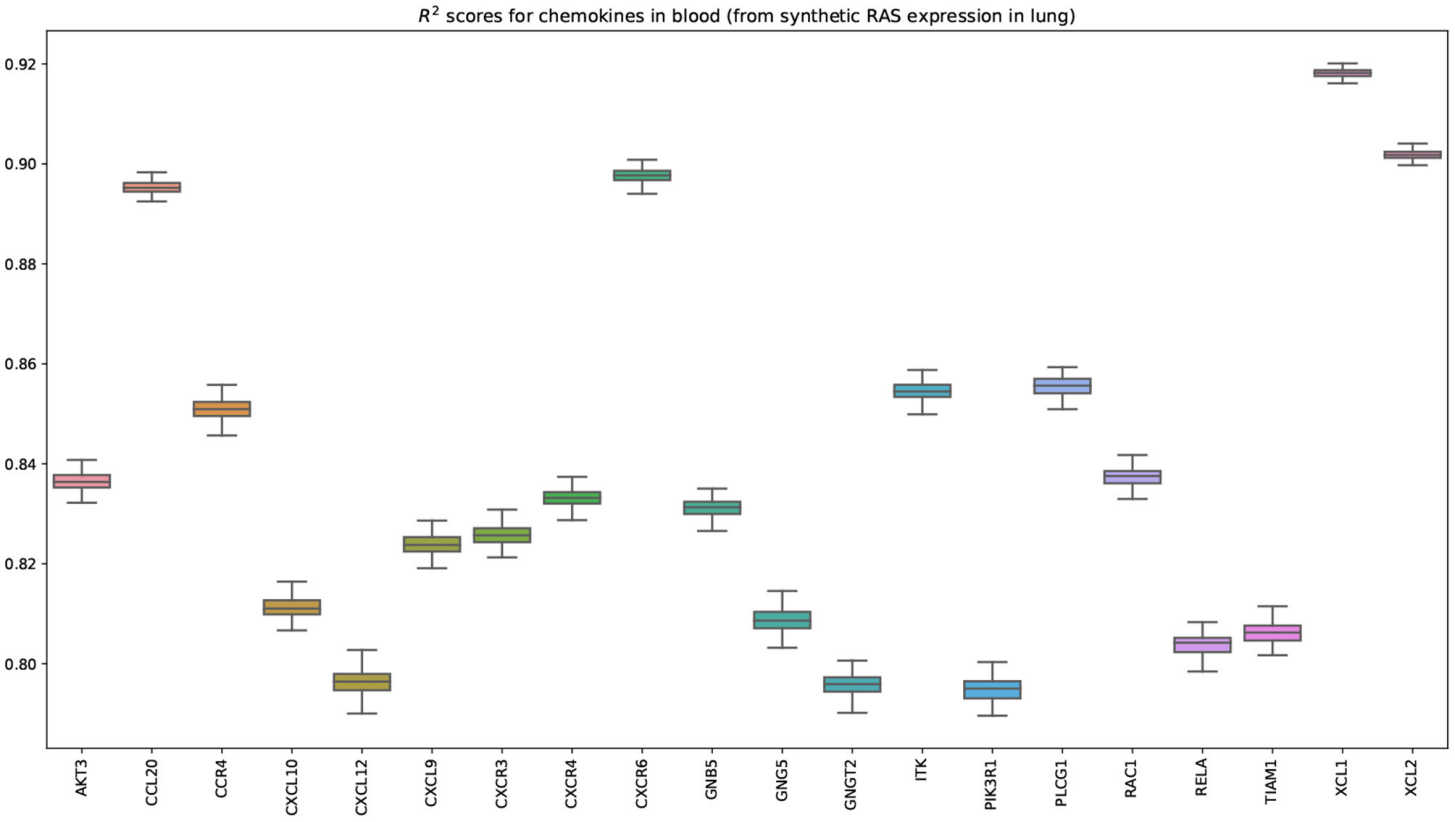
Bootstrapped *R*^2^ scores for chemokines in blood. The input variables are the expressions of 21 genes belonging to the renin-angiotensin system pathway in lung. This plot shows the top 20 predicted chemokines (out of 170). The transcriptomics data was generated by our GAN. Importantly, some of the top predicted chemokines (e.g., CXCR6) have been previously associated with SARS-CoV-2 (Liao et al., 2020).

**Figure 11:**
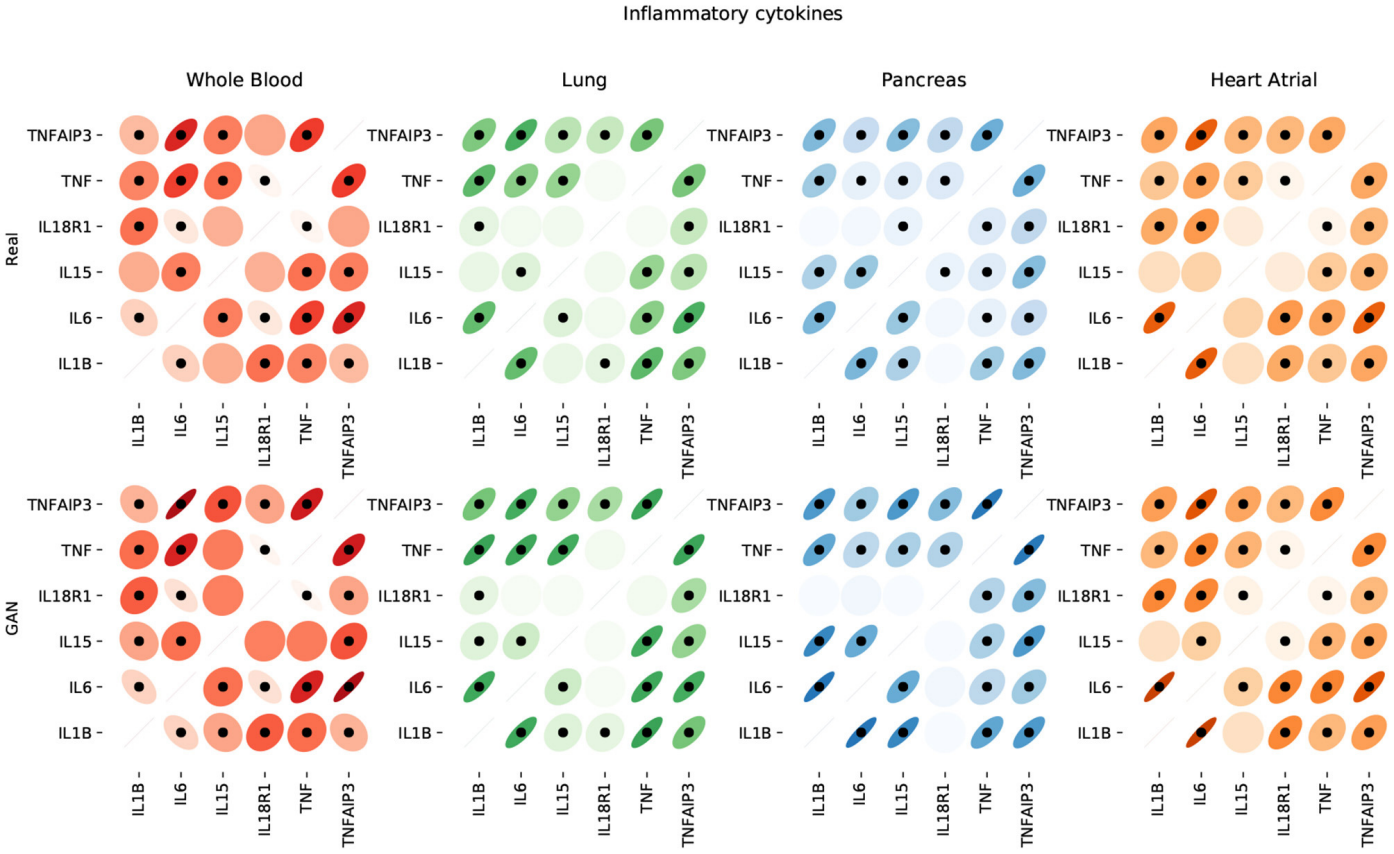
Pairwise correlations between inflammatory cytokines in the 4 modeled tissue types. We use dots to label statistically significant correlations (two-sided *p*-value < 0.05).

**Figure 12:**
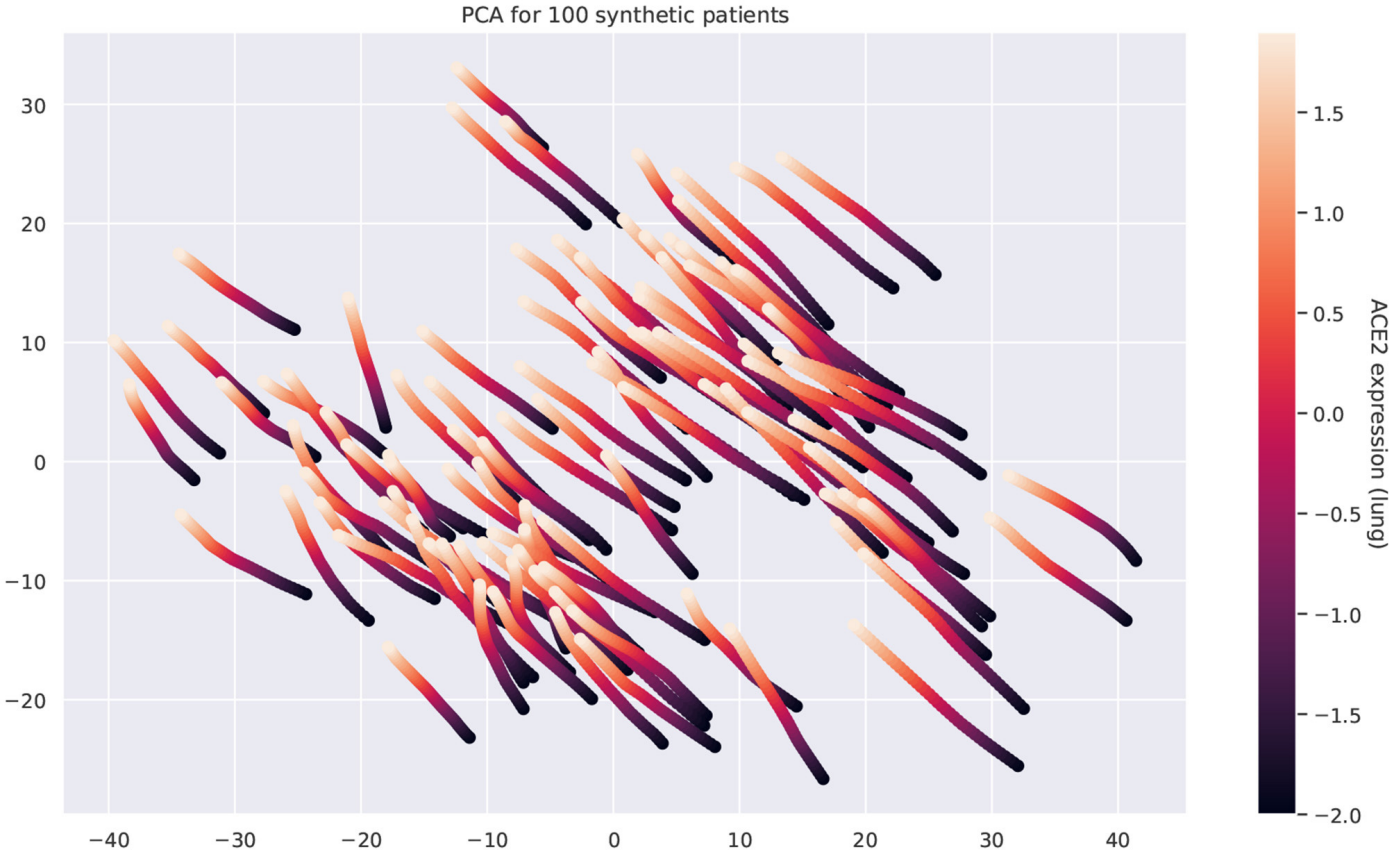
Principal component analysis of the multi-tissue expression of 100 synthetic patients for different levels of ACE2 expression. Each line corresponds to a unique patient. For each patient, we fix all the latent covariates and modify the levels of ACE2 in lung. Overexpressing ACE2 leads to changes in the expression of other genes and these changes follow a well-defined trajectory.

Figure 9 shows that the pairwise correlations between genes in the ACE2 pathway (lung) are well-preserved in the synthetic data. We observe that some genes in the RAS pathway (CTSA, AGTR2, NLN, and PREP) that can be relatively well-explained as a function of blood signaling factors (see Figure 6) are simultaneously correlated with ACE2. This suggests that these genes could be playing an important role in the spread of SARS-CoV-2 in our body through blood. Next, we use the GAN to generate multi-tissue expression data for blood and lung, and fit a linear model to predict the expression of 170 chemokines in blood as a function of the expression of 21 genes in the renin–angiotensin pathway from lung. Figure 10 shows the *R*^2^ scores for the top 20 chemokines. We find that some of the top predicted chemokines (e.g., CXCR6 and XCL1) have previously been associated with SARS-CoV-2 infection (Kusnadi et al., 2020; Liao et al., 2020). Additionally, our GAN captures associations between inflammatory cytokines, which are known to have effects on blood pressure (Groth et al., 2014). Figure 11 illustrates the real and synthetic pairwise correlations for 6 inflammatory cytokines in the 4 modeled tissue types. Finally, Figure 12 shows that it is also possible to sample data for synthetic patients conditioned on different levels of ACE2 expression in lung.

## 4. Discussion

In this work, we presented an interpretable digital twin model providing an holistic view over patients' conditions. We tested our proof of concept on two clinical case studies combining information at organ, tissue, and cellular level showing the potential of our framework in clinical practice. We demonstrate the feasibility of representing and integrating physiological models and molecular information using GNNs and generative adversarial networks. This composite approach provides modularity and scalability across layers of biomedical data, it is amenable of a battery of modeling approaches, and generates integrated predictions that translate into patients trajectories. We have assimilated our product to a digital twin of the patient.

### 4.1. Technological Perspectives

#### 4.1.1. Digital Twin Deployment

Mechanistic computational modeling and machine learning should be considered together when building innovative healthcare solutions. Building a puzzle is often an example of participatory activity. Clinicians, mechanistic computational modeling and machine learning researchers, data policy makers, and public and private sectors could build a puzzle (i.e., the healthcare) together and they should first develop a shared vision about what is the puzzle. Our vision is to consider a co-simulation (say doctor checkup visits vs. computational experiments) of the two twins to allow co-verification. From a theoretical computer science perspective, this could open the direction of an interplay between AI and verification/synthesis and the use of reachability analysis to identify constraints over the well-being and disease system state space. Although different architectures seem suitable (e.g., only GNNs, only GANs, VAEs, etc.), our design has important advantages: the GNN could provide a physical mapping of the human body (in the same way a tube map or bus route is a map of a city); GANs could be specialized on processing molecular information or they could operate cross-modal operations such as omic–omic, omic–clinical, and clinical–clinical.

#### 4.1.2. A Modular Approach

The models presented in this work (GAN and GNN) are independent of each other. On the one hand, the main goal of the GNN model is to forecast various patient's conditions based on real or synthetic data, integrating information that spans multiple layers of the human body. On the other hand, the GAN model is able to generate data under different states, effectively enriching the space of pathophysiological conditions and endowing the digital twin with the ability to simulate the effects of counterfactual events. The independence of these two models enables a modular framework wherein each module can be trained separately on a distinct data modality. Importantly, these modules can be composed and reused through transfer learning. In this work, we have shown how computational models can be used to generate synthetic training data representing physiological conditions. Following the same principles, each module of a complex architecture could be pre-trained on synthetic simulations, refined using data obtained from horizontal population studies, and finally personalized according to clinical health records.

#### 4.1.3. Next-Generation Datasets

The GAN and the GNN models can be interconnected in a synergistic way. In order to train the GNN effectively, it is necessary to have access to heterogeneous, paired data modalities (from different layers: genomic, transcriptomic, cellular, organ, exposomic, etc.) collected from a comprehensive collection of patients and encompassing a wide variety of conditions. However, to the best of our knowledge, to this date no such dataset exists. This is mainly because collecting paired, multilayer data from patients is expensive and entails important ethical and privacy concerns (Jobin et al., 2019; Mittelstadt, 2019). To address this issue, our GAN framework can synthesize data at multiple layers conditioned on the patient's conditions (e.g., diabetic, hypertension, etc.) and clinical information (e.g., heart rate, blood pressure, age, sex, ethnicity, exercise, nutrition, etc.). This synthetic data can be used to train the GNN and impute missing data modalities of real patients. Yet, the lack of real data from patients remains the key limitation for the introduction of our framework in clinical practice.

#### 4.1.4. Explainable AI

The lack of interpretability of deep learning models has been one of the most significant barriers preventing their application in healthcare. Such models exhibit great capacity (Hornik, 1991) but understanding their behavior and following their decision-making process is not trivial (Castelvecchi, 2016). There is a growing body of literature focusing on interpretable artificial intelligence and interpretable deep learning aiming at developing white box models or at explaining black box ones (Das and Rad, 2020). Among such techniques, GNNs have started drawing the attention of both research and industry communities (Bronstein et al., 2017; Zhou et al., 2018). Such models are much more interpretable with respect to other neural approaches thanks to their graph structure, which is quite easy to understand from a human standpoint and a few studies have already shown how graph networks can be effectively employed in biology and healthcare (Zitnik et al., 2018; Gysi et al., 2020).

### 4.2. Advantages, Limitations, and Visions

#### 4.2.1. Toward Precision and Predictive Medicine

The future of medicine is already bound to AI (Topol, 2019). Technological innovations are completely changing medicine perspectives expanding its horizons and moving toward an holistic view of human beings. The destiny of the whole healthcare system depends on this radical paradigm shift. Embracing AI innovations is just a technological prerequisite, and the first step toward a total transformation of how medicine currently works is delivered and perceived by patients. Thinking that AI will just and mainly improve clinical decision making is wrong. AI may actually open the doors to completely new ways of investigating the human body as a whole. The core and ultimate purpose of health will be developing preventative and personalized pathways to well-being rather than delivering treatments. The future foreseen is that AI will assist medicine in improving diagnosis and devising novel therapeutic strategies to deliver more effective solutions. The current healthcare revolution will not take back all the past technological advances, but it will show them under a new light.

#### 4.2.2. Patient's Benefit

A meaningful quote about twins is the following: being a twin is like being born with a best friend. The data integration will make a better portrait of patient's condition trajectories but will require data inter-operability and data security. Technology is often not neutral, but transformed to be biased in one way or another (Ellul et al., 1954). Individuals can have different unforeseen readings and usage of new technologies. It may increase both user vulnerability and user empowerment. The vulnerability is the combination of exposure to the variety of personal medical data and the coping capabilities of users that could be different between young and mature people, as young are usually quicker in incorporating a new technology into everyday life. The user is empowered if he/she acquires awareness and control of his/her condition and context. A common example are online (website and blogs) initiatives such as patientslikeme that allow the user to search and make up his/her mind about a disease (Wicks et al., 2010). Instead, the user disempowerment depends on the lack of technical knowledge of how mechanisms work; this is even enhanced in black box techniques such as deep learning.

#### 4.2.3. Training Clinicians

We believe that improving both data integration and predictability will provide physicians with improved medical decisions support systems and a decrease in both costs, through the evaluation of best therapies, and errors. A limitations is the poor interpretability and explainability in deep learning architectures. This limitation will also greatly affect the training of the new clinicians on AI technologies. There are growing efforts to make neural networks more interpretable in order to keep the human (doctors and patients) in the loop. The interpretability could be improved by using parallel mechanistic computational modeling and simulations (Milanesi et al., 2009; Bartocci and Lió, 2016), model extraction libraries (see, for instance, Kazhdan et al., 2020), and visual inference tools (Bodnar et al., 2020). This tool could also be complemented by clinical decision support systems such as Müller and Lio (2020). The complexly structured and multilevel comorbidity and frailty patterns of most diseases describe a highly dynamical system and are, therefore, challenging current medical therapies.

#### 4.2.4. AI for Evidence-Based Medicine

From a clinical standpoint, AI will support a plethora of different tasks from medical check up to personalized intervention strategies to contrast ripple effects or to promote healthy habits. In non-acute states, predictive inference will propose prevention plans for comorbidity management, particularly in presence of multiple therapies (Rivera, 2020). Increasingly large amount of personal data will be collected to feed modular machine learning (ML) models organized to address specific and personalized medical issues. Clinical endpoints will be constantly monitored, shared, and compared in order to answer relevant research questions and to deliver the best possible service. A deeper understanding and practice of modeling in medicine will produce better investigation of complex biological processes, and even new ideas and better feedback into medicine. Modeling-based approaches combined with data-driven ML techniques will progressively provide models with higher degree of interpretability and generalization ability (Barbiero et al., 2020a), which will make evidence-based medicine even more accessible intensifying the involvement of patients in the decision-making process. AI simulations forecasting the evolution of clinical endpoints over time will also reshape clinical guidelines (Rivera, 2020), which will no longer be based just on *horizontal* population studies. Cross-modality data will be collected for each patient and machine learning models will be used to predict a bundle of possible trajectories representing the future states of the patient allowing for personalized prescriptions, surgical planning, and medical interventions.

#### 4.2.5. Social Impact

Ethical repercussions will also be huge (Jobin et al., 2019; Mittelstadt, 2019). The transition will call for deeper trans-disciplinary research and a substantial technological innovation in a variety of research and social areas. Here, education will play a key role in changing lifestyle habits and the way health is perceived, communicated, and delivered (Yu et al., 2017). For each individual, both healthcare systems and private companies will collect, save, and eventually exploit an enormous amount of personal data. Providing an effective, stable, and unified juridical overview is critical on this matter (Panch et al., 2019).

#### 4.2.6. Next-Generation Medical Devices

AI will change the leading *vehicle* of medicine. The demand for AI-powered and internet of things (IoT) devices is increasing worldwide. The future equipment for precision medicine will likely required to be cheap and extremely modular, but more importantly it needs to be deployable in dedicated hardware to be distributed in larger markets. Our digital twin model aims at providing the first example of a novel class of AI-assisted tools for precision and predictive medicine. Our framework is designed to scale to medical device deployment and run time monitoring and verification combining ideas from systems medicine with scientific computing and machine learning. The integration of interpretable AI models in clinical devices may lead to a deep transformation in healthcare paving the way for a next generation of tools for precision medicine probing the inner workings of full body in well-being and disease conditions.

## 5. Methods

### 5.1. Graph Neural Network

#### 5.1.1. Graph Network Blocks

The GNN framework proposed by Battaglia et al. (2018) is based on modules called graph network blocks (GN blocks) representing the core computation units of a GNN. Multiple GN blocks can be composed of or even combined with other neural networks to generate complex architectures. A GNN can be defined as a 3-tuple *G* = (**u**, *H, E*). $H={\left\{{\text{h}}_{i}\right\}}_{i=1:N^{v}}$ is the node set where the feature of each node is denoted by **h**_*i*_. *E* = {(**e**_*k*_, *r*_*k*_, *s*_*k*_)} is the edge set where each node is represented by its features **e**_*k*_, the receiver node *r*_*k*_, and the sender node *s*_*k*_. **u** denotes a set of global attributes representing the state of the underlying system. Each GN block consists of three update functions, ϕ, and three aggregation functions, ρ:


$$\begin{array}{l} {\text{e}}_{k}^{\prime }=\phi^{e}\left({\text{e}}_{k},{\text{h}}_{r_{k}},{\text{h}}_{s_{k}},\text{u}\right)\;{\bar{\text{e}}}_{i}^{\prime }=\rho^{e\to h}\left(E_{i}^{\prime }\right) \end{array}$$

(1) $$\begin{array}{l} {\text{h}}_{i}^{\prime }=\phi^{h}\left({\bar{\text{e}}}_{k},{\text{h}}_{i},\text{u}\right)\;{\bar{\text{e}}}^{\prime }=\rho^{e\to u}\left(E^{\prime }\right) \end{array}$$

$$\begin{array}{l} {\text{u}}^{\prime }=\phi^{u}\left({\text{e}}^{\prime },{\text{h}}^{\prime },\text{u}\right)\;{\bar{\text{h}}}^{\prime }=\rho^{h\to u}\left(H^{\prime }\right) \end{array}$$

where $E_{i}^{\prime }=\left\{\left({\text{e}}_{k}^{\prime },r_{k},s_{k}\right)\right\}$, $H^{\prime }={\left\{\left({\text{h}}_{i}^{\prime }\right)\right\}}_{i=1:N^{v}}$, and $E^{\prime }={⋃}_{i}E_{i}^{\prime }={\left\{\left({\text{e}}_{k}^{\prime },r_{k},s_{k}\right)\right\}}_{k=1:N^{e}}$. In order to train a GN block in full, six computation steps are required, alternating the update and aggregation functions. For each edge, $E_{i}^{\prime }$ is computed through the update function ϕ^*e*^. The result is then aggregated by means of the function ρ^*e*→*v*^. The output ${\bar{\text{e}}}_{i}^{\prime }$ corresponds to an edge update and it is employed to update node representations ${\text{h}}_{i}^{\prime }$ by means of ϕ^*h*^ . ρ^*e*→*u*^ and ρ^*h*→*u*^ perform aggregation steps generating ${\bar{\text{e}}}^{\prime }$ and ${\bar{\text{h}}}^{\prime }$ from edge and node updates, respectively. Global attributes represented by **u**′ are computed leveraging the information from ${\bar{\text{e}}}^{\prime }$, ${\bar{\text{h}}}^{\prime }$, and **u** via the function ϕ^*u*^. The learning process of each GN block may be independent or co-dependent with other blocks. Constraints may apply on edges, information flows, or global attributes, depending on the application. In this work, we are just interested in the evaluation of global attributes to monitor clinical endpoints and we did not apply any learning constraint, even if in clinical practice may still be of great interest. Given a set of labels for global attributes $\text{t}={\left\{t_{i}\right\}}_{i=1:N^{v}}$ and the corresponding predictions provided by the GN block ${\hat{\text{u}}}^{\prime }={\left\{{\hat{u}}_{i}^{\prime }\right\}}_{i=1:N^{v}}$ representing the evolution of the underlying biological system, we aim at minimizing the following objective function:


(2) $$\begin{array}{l} \underset{\theta }{\min}\frac{1}{N^{v}}\sum_{i=1}^{N^{v}}{\left(t_{i}-{\hat{u}}_{i^{\prime }}\right)}^{2} \end{array}$$

where θ is the set of model's parameters.

#### 5.1.2. Assessing Prediction Uncertainty

The aim of developing a digital patient model is to provide an accurate estimation of the trajectory of a patient by forecasting clinically relevant endpoints. In such a context, quantifying model uncertainty is critical. One of the most established techniques relies upon the use of dropout (Srivastava et al., 2014) at test time, as a Bayesian approximation, without sacrificing either computational complexity or test performance (Gal and Ghahramani, 2016b). In this framework, the first two moments of the predictive distribution *q* performing *T* stochastic forward passes for a sample x^*^ with label y^*^ can be estimated as (Gal and Ghahramani, 2016a):


(3) $$\begin{array}{lll} {𝔼}_{q\left({\text{y}}^{*},{\text{x}}^{*}\right)}\left({\text{y}}^{*}\right) & \approx & \frac{1}{T}\overset{T}{\underset{i=1}{\sum }}{\hat{\text{y}}}^{*}\left({\text{x}}^{*},W_{1}^{t},\ldots ,W_{L}^{t}\right) \end{array}$$

$$\begin{array}{rcl} {\text{Var}}_{q\left({\text{y}}^{*},{\text{x}}^{*}\right)}\left({\text{y}}^{*}\right) & \approx & \tau^{-1}I_{D}\\ + & \frac{1}{T}\overset{T}{\underset{i=1}{\sum }}{\hat{\text{y}}}^{*}{\left({\text{x}}^{*},W_{1}^{t},\ldots ,W_{L}^{t}\right)}^{T}{\hat{\text{y}}}^{*}\left({\text{x}}^{*},W_{1}^{t},\ldots ,W_{L}^{t}\right) \end{array}$$

(4) $$\begin{array}{rc} - & E_{q\left({\text{y}}^{*},{\text{x}}^{*}\right)}{\left({\text{y}}^{*}\right)}^{T}E_{q\left({\text{y}}^{*},{\text{x}}^{*}\right)}\left({\text{y}}^{*}\right) \end{array}$$

where ${\hat{\text{y}}}^{*}$ is the predicted label, ${\left\{W_{i}\right\}}_{i=1}^{L}$ is a set of random variables representing the weights of a neural network with *L* layers, *I*_*D*_ is an identity matrix, *D* is the number of output units of the neural network, and τ is a precision hyper-parameter. The method has also been generalized to convolutional (Gal and Ghahramani, 2015) and recurrent networks (Gal and Ghahramani, 2016c).

Here, we show how such technique can be used to quantify the uncertainty of a GNN by generating a predictive distribution of the trajectories representing the future states of the patient. Let $x_{1}^{*},\ldots ,x_{k}^{*}$ be a sequence of real values representing a clinical endpoint measured at 1, …, *k* time steps. Let *f*^*t*^ be a stochastic model that takes a sequence $x_{1}^{*},\ldots ,x_{k}^{*}$ as input and it outputs a prediction ${\hat{y}}^{*}\in \mathbb{R}$. We are interested in estimating a predictive distribution of the trajectories of the variable *x* over the next *k* + 1, …, *k* + *h* time steps. To this aim, we can use an iterative algorithm by generating one trajectory at a time. The first prediction ${\hat{y}}_{k+1}^{*}$ can be generated as:


(5) $$\begin{array}{l} {\hat{y}}_{k+1}^{*,t}=f^{t}\left(x_{1}^{*},\ldots ,x_{k}^{*}\right) \end{array}$$

By using the obtained prediction and sliding the time window one time step further, we can generate the first prediction for the second time step *k* + 2:


(6) $$\begin{array}{l} {\hat{y}}_{k+2}^{*,t}=f^{t}\left(x_{2}^{*},\ldots ,x_{k}^{*},{\hat{y}}_{k+1}^{*,t}\right) \end{array}$$

The procedure can be repeated for *k* + *h* time steps to generate a single trajectory. Model uncertainty can be assessed building multiple trajectories by performing *T* stochastic forward passes. The resulting algorithm is equivalent to a Monte Carlo sampling as proven by Gal and Ghahramani (2016b). In our GNN model, the approach we just described can be easily applied for each node in order to assess the uncertainty of clinical endpoints.

### 5.2. Generative Adversarial Network

Consider a dataset D={(x,m,r,q)} of samples from an unknown distribution ℙ_**x**, **m**, **r**, **q**_, where **x** ∈ ℝ^*t*×*n*^ represents a matrix of *n* gene expression values in *t* tissues; **m** ∈ {0, 1}^*t*^ is a mask vector indicating whether the expression of each tissue has been measured for the given patient; and **r** ∈ ℝ^*k*^ and **q** ∈ ℕ^*c*^ are vectors of *k* quantitative covariates (e.g., age) and *c* categorical (e.g., gender), respectively. Our goal is to produce realistic gene expression samples by modeling the conditional probability distribution ℙ(**X** = **x**|**M** = **m**, **R** = **r**, **Q** = **q**), where **r** includes the expression of ACE2 in different tissues (e.g., lung, kidney, and pancreas). By modeling this distribution, we can sample data for different conditions and quantify the uncertainty of the generated expression values.

To address this problem, we extend the model proposed in Viñas et al. (2021) to simultaneously account for *t* tissue types from the same donor. In particular, our method builds on a Wasserstein GAN with gradient penalty (WGAN-GP) (Arjovsky et al., 2017; Gulrajani et al., 2017). Similar to Generative Adversarial Networks (GAN) (Goodfellow et al., 2014), WGAN-GPs estimate a generative model via an adversarial process driven by the competition between two players, the *generator* and the *critic*.

The generator aims at producing samples from the conditional ℙ(**X**|**M**, **R**, **Q**). Formally, we define the generator as a function $G_{\theta }:{\mathbb{R}}^{u}\times {\mathbb{R}}^{k}\times {\mathbb{N}}^{c}\to {\mathbb{R}}^{t\times n}$ parameterized by θ that generates gene expression values $\hat{\text{x}}$ as follows:


(7) $$\begin{array}{l} \hat{\text{x}}=\text{m}⊙G_{\theta }\left(\text{z},\text{r},\text{q}\right) \end{array}$$

where **z** ∈ ℝ^*u*^ is a vector sampled from a fixed noise distribution ℙ_**z**_ and *u* is a user-definable hyperparameter. We apply the mask **m** element-wise to match the distribution of missing tissues of the training dataset.

The critic takes gene expression samples **x** from two input streams (the generator and the data distribution) and attempts to distinguish the true input source. Formally, the critic is a function $D_{\omega }:{\mathbb{R}}^{t\times n}\times {\left\{0,1\right\}}^{t}\times {\mathbb{R}}^{k}\times {\mathbb{N}}^{c}\to \mathbb{R}$ parameterized by ω that we define as follows:


$$\begin{array}{l} ȳ=D_{\omega }\left(\bar{\text{x}},\text{m},\text{r},\text{q}\right) \end{array}$$

where the output ȳ is an unbounded scalar that quantifies the degree of realism of an input sample $\bar{\text{x}}$ given the covariates **r** and **q** (e.g., high values correspond to real samples and low values correspond to fake samples). When the expression of a certain tissue *t* is unavailable for a given patient, we set the unobserved values of tissue *t* in $\bar{\text{x}}$ to 0 and the *t*-th component of the mask **m** to 0.

We optimize the generator and the critic adversarially. Following (Arjovsky et al., 2017), we train the generator *G*_θ_ and the critic *D*_ω_ to solve the following minimax game based on the Wasserstein distance:


(8) $$\begin{array}{l} \underset{\theta }{\min}\;\underset{\omega }{\min}\underset{x,m,r,q\sim {\mathbb{P}}_{x,m,r,q}}{E}\left[D_{\omega }(x,m,r,q)-\underset{z\sim {\mathbb{P}}_{z}}{E}[D_{\omega }(\hat{x},m,r,q)]\right]\\ \text{subject to }||D_{\omega }(x_{i},m,r,q)-D_{\omega }(x_{j},m,r,q)||\leq ||x_{i}-x_{j}||\\ \;\forall x_{i},x_{j}\in {\mathbb{R}}^{t\times n},\;m\in {\left\{0,1\right\}}^{t},r\in {\mathbb{R}}^{k},q\in {\mathbb{N}}^{c} \end{array}$$

where $\hat{\text{x}}$ is defined as in Equation (7) and the constraint enforces a soft version of the 1-Lipschitz constraint (e.g., the norm of the critic's gradient with respect to **x** must be at most 1 everywhere).

Let ${\left\{\left({\text{x}}_{i},{\text{m}}_{i},{\text{r}}_{i},{\text{q}}_{i}\right)\right\}}_{i=1}^{b}$ be a mini-batch of *b* independent samples from the training dataset D. Let {**z**_1_, **z**_2_, ..., **z**_*k*_} be a set of *k* vectors sampled independently from the noise distribution ℙ_**z**_ and let us define the synthetic samples corresponding to the mini-batch as ${\hat{\text{x}}}_{i}={\text{m}}_{i}⊙G_{\theta }\left({\text{z}}_{i},{\text{r}}_{i},{\text{q}}_{i}\right)$ for each *i* in [1, 2, ..., *k*]. We solve the minimax problem described in Equation (8) by interleaving mini-batch gradient updates for the generator and the critic, optimizing the following problems:


(9) $$\begin{array}{l} \text{Generator: }\underset{\theta }{\min}\;-\frac{1}{k}\sum_{i=1}^{k}D_{\omega }\left({\hat{x}}_{i},m_{i},r_{i},q_{i}\right)\\ \text{Critic: }\underset{\omega }{\min}\;\frac{1}{k}\sum_{i=1}^{k}D_{\omega }\left({\hat{x}}_{i},m_{i},r_{i},q_{i}\right)-D_{\omega }\left(x_{i},m_{i},r_{i},q_{i}\right)\\ \;+\frac{\lambda }{k}\sum_{i=1}^{k}{\left(||\nabla_{{\tilde{x}}_{i}}D_{\omega }({\tilde{x}}_{i},m_{i},r_{i},q_{i})|{|}_{2}-1\right)}^{2} \end{array}$$

where λ is a user-definable hyperparameter and each ${\tilde{\text{x}}}_{i}$ is a random point along the straight line that connects **x**_*i*_ and ${\hat{\text{x}}}_{i}$, that is, ${\tilde{\text{x}}}_{i}=\alpha_{i}{\text{x}}_{i}+\left(1-\alpha_{i}\right){\hat{\text{x}}}_{i}$ with $\alpha_{i}\sim U\left(0,1\right)$. Intuitively, since enforcing the 1-Lipschitz constraint everywhere is intractable (see Equation 8), the second term of the critic problem is a relaxed version of the constraint that penalizes the gradient norm along points in the straight lines that connect real and synthetic samples (Gulrajani et al., 2017).

#### 5.2.1. Architecture

Figure 1 shows the architecture of both players. The generator *G* receives a noise vector **z** as input (green box) as well as sample covariates **r** and **q** (orange boxes) and produces a vector $\hat{\text{x}}$ of synthetic expression values (red box). The critic *D* takes either a real gene expression sample **x** (blue box) or a synthetic sample $\hat{\text{x}}$ (red box), in addition to sample covariates **r** and **q**, and attempts to distinguish whether the input sample is real or fake. For both players, we use word embeddings (Mikolov et al., 2013) to model the sample covariates (light green boxes), a distinctive feature that allows to learn distributed, dense representations for the different tissue types and, more generally, for all the categorical covariates **q** ∈ ℕ^*c*^.

Formally, let *q*_*j*_ be a categorical covariate (e.g., tissue type) with vocabulary size *v*_*j*_, that is, *q*_*j*_ ∈ {1, 2, ..., *v*_*j*_}, where each value in the vocabulary {1, 2, ..., *v*_*j*_} represents a different category (e.g., whole blood or kidney). Let ${\bar{\text{q}}}_{j}\in {\left\{0,1\right\}}^{v_{j}}$ be a one-hot vector such that ${\bar{q}}_{jk}=1$ if *q*_*j*_ = *k* and ${\bar{q}}_{jk}=0$ otherwise. Let *d*_*j*_ be the dimensionality of the embeddings for covariate *j*. We obtain a vector of embeddings ${\text{e}}_{j}\in {\mathbb{R}}^{d_{j}}$ as follows:


$$\begin{array}{l} {\text{e}}_{j}={\text{W}}_{j}{\bar{\text{q}}}_{j} \end{array}$$

where each ${\text{W}}_{j}\in {\mathbb{R}}^{d_{j}\times v_{j}}$ is a matrix of learnable weights. Essentially, this operation describes a lookup search in a dictionary with *v*_*j*_ entries, where each entry contains a learnable *d*_*j*_-dimensional vector of embeddings that characterizes each of the possible values that *q*_*j*_ can take. To obtain a global collection of embeddings **e**, we concatenate all the vectors **e**_*j*_ for each categorical covariate *j*:


$$\begin{array}{l} e=\|{\;}_{j=1}^{c}e_{j} \end{array}$$

where *c* is the number of categorical covariates and ∥ represents the concatenation operator. We then use the learnable embeddings **e** in downstream tasks.

In terms of the player's architecture, we model both the generator *G*_θ_ and critic *D*_ω_ as neural networks that leverage independent instances **e**^*G*^ and **e**^*D*^ of the categorical embeddings for their corresponding downstream tasks. Specifically, we model the two players as follows:


$$\begin{array}{l} G_{\theta }\left(\text{z},\text{r},\text{q}\right)=\text{MLP}\left(\text{z}∥\text{r}∥{\text{e}}^{G}\right)\;D_{\omega }\left(\bar{\text{x}},\text{m},\text{r},\text{q}\right)=\text{MLP}\left(\bar{\text{x}}∥\text{m}∥\text{r}∥{\text{e}}^{D}\right) \end{array}$$

where MLP denotes a multilayer perceptron.

### 5.3. Ridge Regression

We model the expression of genes from the renin-angiotensin system in lung, kidney, pancreas, and heart as a function of genes in the chemokine, TNF, and TGF-β pathways in blood. Let $\text{Y}={\left(Y_{1},...,Y_{n}\right)}^{⊤}$ and $\text{X}={\left(X_{1},...,X_{m}\right)}^{⊤}$ be multivariate random variables representing the expression of the *n* genes in the renin-angiotensin system and the *m* genes in the signaling pathways, respectively. Our model is based on ridge regression (Hoerl and Kennard, 1970):


$$\begin{array}{l} \text{Y}=\text{X}\text{W}+\epsilon \end{array}$$

where **W** ∈ ℝ^*m*×*n*^ is a matrix of learnable weights and **ϵ** ∈ ℝ^*n*^ are the residuals. We optimize the following objective:


$$\begin{array}{l} \underset{\text{W}}{\min}||\text{Y}-\text{X}\text{W}{||}_{2}^{2}+\alpha ||\text{W}{||}_{2}^{2} \end{array}$$

where α is a hyperparameter that controls the regularization strength. Alternative non-linear models such as support vector machines, Gaussian processes, and random forests did not improve our cross-validation scores.

## Data Availability Statement

We leverage data from the Genotype-Tissue Expression (GTEx) project (v8), a resource that has generated a comprehensive collection of human transcriptome data in a diverse set of tissues (Aguet et al., 2019). We use the KEGG pathway database (Kanehisa et al., 2010) to select genes from the renin-angiotensin system (*hsa04614*), chemokine (*hsa04062*), TNF (*hsa04668*), and TGF-β (*hsa04350*) pathways.

The computational ODE-based system described in Barbiero and Lió (2020) is used to generate a time series for each differential equation with a window size of τ = 500 time steps (Barbiero and Lio, 2020). Time series are collected, randomly shuffled, and stacked in a dataset. Each item of the collection is randomly assigned either to a training (*n*_*train*_ = 3200), validation (*n*_*val*_ = 800), or test set (*n*_*test*_ = 1000).

## Code availability

All the code for the experiments has been implemented in Python 3, relying upon open-source libraries (Pedregosa et al., 2011; Abadi et al., 2016; Wang et al., 2019). All the experiments have been run on the same machine: Intel® Core™ i7-8750H 6-Core Processor at 2.20 GHz equipped with 8 GB RAM. To enable code reuse, the Python code for the mathematical models including parameter values and documentation is freely available under GNU Public License from a GitHub repository^1^ (Barbiero et al., 2020b). Unless required by applicable law or agreed to in writing, software is distributed on an “as is” basis, without warranties or conditions of any kind, either express or implied.

## Author Contributions

PB and RV performed the experiments. All authors were involved in conceiving the approach, co-wrote the paper, designing the experiments, reviewing and discussing the data, and commented on the manuscript.

## Funding

This project has received funding from ‘‘la Caixa'' Foundation (ID 100010434), under agreement 667 LCF/BQ/EU19/11710059, and from the Engineering and Physical Sciences 668 Research Council under grant agreement No EP/R513180/1 (R.V. EPSRC DTG 2018/19).

## Conflict of Interest

The authors declare that the research was conducted in the absence of any commercial or financial relationships that could be construed as a potential conflict of interest.

## Publisher's Note

All claims expressed in this article are solely those of the authors and do not necessarily represent those of their affiliated organizations, or those of the publisher, the editors and the reviewers. Any product that may be evaluated in this article, or claim that may be made by its manufacturer, is not guaranteed or endorsed by the publisher.

## Footnotes

^1^ https://github.com/pietrobarbiero/digital-patient
