# Supplementary material for: Graph Representation Forecasting of Patient's Medical Conditions: Toward a Digital Twin
Source: Front Genet. 2021 Sep 16;12:652907. doi: 10.3389/fgene.2021.652907 (PMC8481902; doi:10.3389/fgene.2021.652907)
Supplement: Supplementary file 1 [file Data_Sheet_1.PDF]

frontiers

Supplementary Material

1 GNN HYPERPARAMETERS

The graph neural model we used in our experiments is a Graph Attention Network (Veličković et al., 2017) with two graph convolutional layers (50 and 10 units respectively) with rectified linear unit (ReLU) activations. To optimise the model, we use minibatch gradient-descent (with a batch size of 128) and the Adam optimiser with decoupled weight decay (Loshchilov and Hutter, 2017) with a learning rate of 0.005. We trained for a maximum of 1000 epochs saving the model generating the lowest loss on a validation set. The ranges of hyperparameters analyzed for the GNN are presented in Figure S1.

Table S1. Hyper parameter ranges for the GNN model.

	learning rate	max epochs	hidden neurons
min	10^{-4}	500	[50, 20]
max	10^{-2}	1000	[100, 40]

2 GAN HYPERPARAMETERS

2.1 Hyperparameters

We model both the generator and critic as a fully neural network with one hidden layer (64 units) and a rectified linear unit (ReLU) activation. For the generator, we use a latent dimension of 32 units. We include the sex and the patient cohort as categorical covariates and set the embedding dimensions as $\lceil\sqrt{v}\rceil$ where v is the vocabulary size of each category. We include the age of the patient as a numerical covariate.

To optimise the model, we use minibatch gradient-descent (with a batch size of 32) and the RMSprop optimiser (Hinton et al., 2012) with a learning rate of 0.0005. We use a gradient penalty weight λ of 10. We train the model for a maximum of 5000 epochs and save the weights that maximise the evaluation metric defined in the following section for a separate holdout set.

2.2 Evaluation metric

Let $\mathbf{A}$ be a $n \times n$ symmetric matrix holding the pairwise distances between n genes. In order to measure how faithfully this matrix preserves the pairwise distances with respect to another $n \times n$ distance matrix $\mathbf{B}$, we define the Pearson's correlation coefficient between the elements in the upper-diagonal of $\mathbf{A}$ and $\mathbf{B}$:

$$\gamma(\mathbf{A}, \mathbf{B}) = \sum_{i=1}^{n-1} \sum_{j=i+1}^{n} \left(\frac{A_{i,j} - \mu(\mathbf{A})}{\sigma(\mathbf{A})}\right)\left(\frac{B_{i,j} - \mu(\mathbf{B})}{\sigma(\mathbf{B})}\right)$$

where, for a given $n \times n$ matrix $\mathbf{G}$, $\mu(\mathbf{G})$ and $\sigma(\mathbf{G})$ are defined as:

$$\mu(\mathbf{G}) = \frac{2}{n(n-1)} \sum_{i=1}^{n-1} \sum_{j=i+1}^{n} G_{i,j}$$

$$\sigma(\mathbf{G}) = \sqrt{\frac{2}{n(n-1)} \sum_{i=1}^{n-1} \sum_{j=i+1}^{n} (G_{i,j} - \mu(\mathbf{G}))^2}$$

Distance between *real* and *artificial* distance matrices. Let $\mathbf{X} \in \mathbb{R}^{m_1 \times n}$ and $\mathbf{Z} \in \mathbb{R}^{m_2 \times n}$ be two matrices containing m_1 real and m_2 synthetic expression observations for n genes, respectively. For a given distance function d (we use the Pearson's dissimilarity coefficient), we define two $n \times n$ distance matrices $\mathbf{D}^X$ and $\mathbf{D}^Z$ as:

$$D^X_{i,j} = d(col(\mathbf{X}, i), col(\mathbf{X}, j)) \quad D^Z_{i,j} = d(col(\mathbf{Z}, i), col(\mathbf{Z}, j)) \tag{S1}$$

where $col(\mathbf{X}, i)$ is the i-th column of matrix $\mathbf{X}$.

The coefficient $\gamma(\mathbf{D}^X, \mathbf{D}^Z) \in [-1, 1]$ measures whether the pairwise distances between genes from the real data are correlated with those from the synthetic data.

2.3 Robustness across number of layers and units

Figure S1 shows the evaluation scores across steps for several configurations of the generator and critic architectures. We note that increasing the number of layers does not yield better scores (i.e. the maximum scores are achieved when using 1 hidden layer) and that the model is fairly robust across different hyperparameter choices.

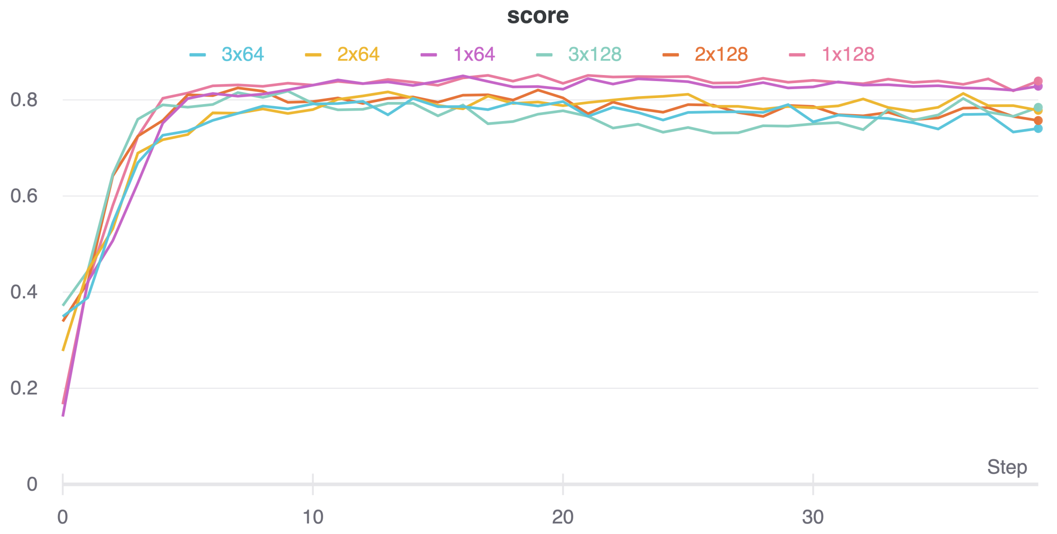

Figure S1. Evaluation score $\gamma(\mathbf{D}^X, \mathbf{D}^Z)$ across steps for several configurations of the generator and critic architectures. The labels indicate the number of hidden layers and units per layer, respectively. For example, for 2x64, the MLPs of both the generator and critic consist of 2 hidden layers with 64 units per layer. Each step is 5 training epochs. Increasing the number of layers does not yield better scores. Additionally, we note that the model is fairly robust across different hyperparameter choices.

3 REGULARISATION STRENGTH OF RIDGE REGRESSION

Figure S2 shows the held-out performances across different values of α, the regularisation hyperparameter of ridge regression, for the model employed in Section 3.3. We note that the held-out scores are quite stable after reaching a certain threshold.

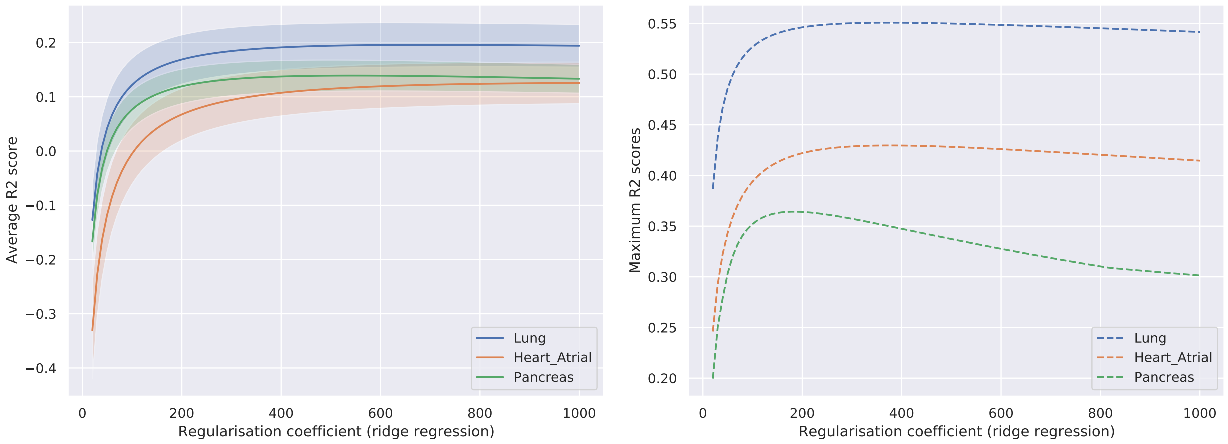

Figure S2. Average and maximum R^2 scores across regularisation strength (ridge regression) and tissues. For the left plot, we average the per-gene scores across genes in the renin-angiotensin system pathway (RAS). The shaded area represents the standard error. The relatively small scores can be explained by the fact that the expression of several RAS genes cannot be inferred from signalling genes in whole blood (see Figure 7). The right plot shows the maximum R^2 scores (corresponding to the best predicted RAS gene).

REFERENCES

Hinton, G., Srivastava, N., and Swersky, K. (2012). Neural networks for machine learning lecture 6a overview of mini-batch gradient descent 14

Loshchilov, I. and Hutter, F. (2017). Decoupled weight decay regularization. *arXiv preprint arXiv:1711.05101*

Veličković, P., Cucurull, G., Casanova, A., Romero, A., Lio, P., and Bengio, Y. (2017). Graph attention networks. *arXiv preprint arXiv:1710.10903*
